# Dehydrogenase *versus* oxidase function: the interplay between substrate binding and flavin microenvironment

**DOI:** 10.1021/acscatal.4c05944

**Published:** 2025-01-02

**Authors:** Teresa Benedetta Guerriere, Alessandro Vancheri, Ilaria Ricotti, Stefano A Serapian, Daniel Eggerichs, Dirk Tischler, Giorgio Colombo, Maria L. Mascotti, Marco W. Fraaije, Andrea Mattevi

**Affiliations:** 1 Department of Biology and Biotechnology “Lazzaro Spallanzani”, University of Pavia, Pavia, Italy 27100; 2 Department of Chemistry, University of Pavia, 27100 Pavia, Italy; 3 Microbial Biotechnology, Ruhr University Bochum, 44780, Bochum, Germany; 4 IHEM CONICET, Universidad Nacional de Cuyo, Mendoza, Argentina, M5502JMA; 5 Molecular Enzymology Group, University of Groningen, Groningen, The Netherlands, 9747AG

**Keywords:** enzyme evolution, flavin, oxygen biochemistry, oxidation, oxidative metabolism, microbial metabolism

## Abstract

Redox enzymes, mostly equipped with metal or organic cofactors, can vary their reactivity with oxygen by orders of magnitudes. Understanding how oxygen reactivity is controlled by the protein milieu remains an open issue with broad implications for mechanistic enzymology and enzyme design. Here, we address this problem by focusing on a widespread group of flavoenzymes that oxidize phenolic compounds derived from microbial lignin degradation, using either oxygen or a cytochrome c as electron acceptors. A comprehensive phylogenetic analysis revealed conserved amino acid motifs in their flavin-binding site. Using a combination of kinetics, mutagenesis, structural, and computational methods, we examined the role of these residues. Our results demonstrate that subtle and localized changes in the flavin environment can drastically impact on oxygen reactivity. These effects are afforded through the creation or blockade of pathways for oxygen diffusion. Substrate binding plays a crucial role by potentially obstructing oxygen access to the flavin, thus influencing the enzyme’s reactivity. The switch between oxidase and dehydrogenase functionalities is thereby achieved through targeted, site-specific amino acid replacements that finely tune the microenvironment around the flavin. Our findings explain how very similar enzymes can exhibit distinct functional properties, operating as oxidases or dehydrogenases. They further provide valuable insights for the rational design and engineering of enzymes with tailored functions.

## Introduction

**Scheme 1:**
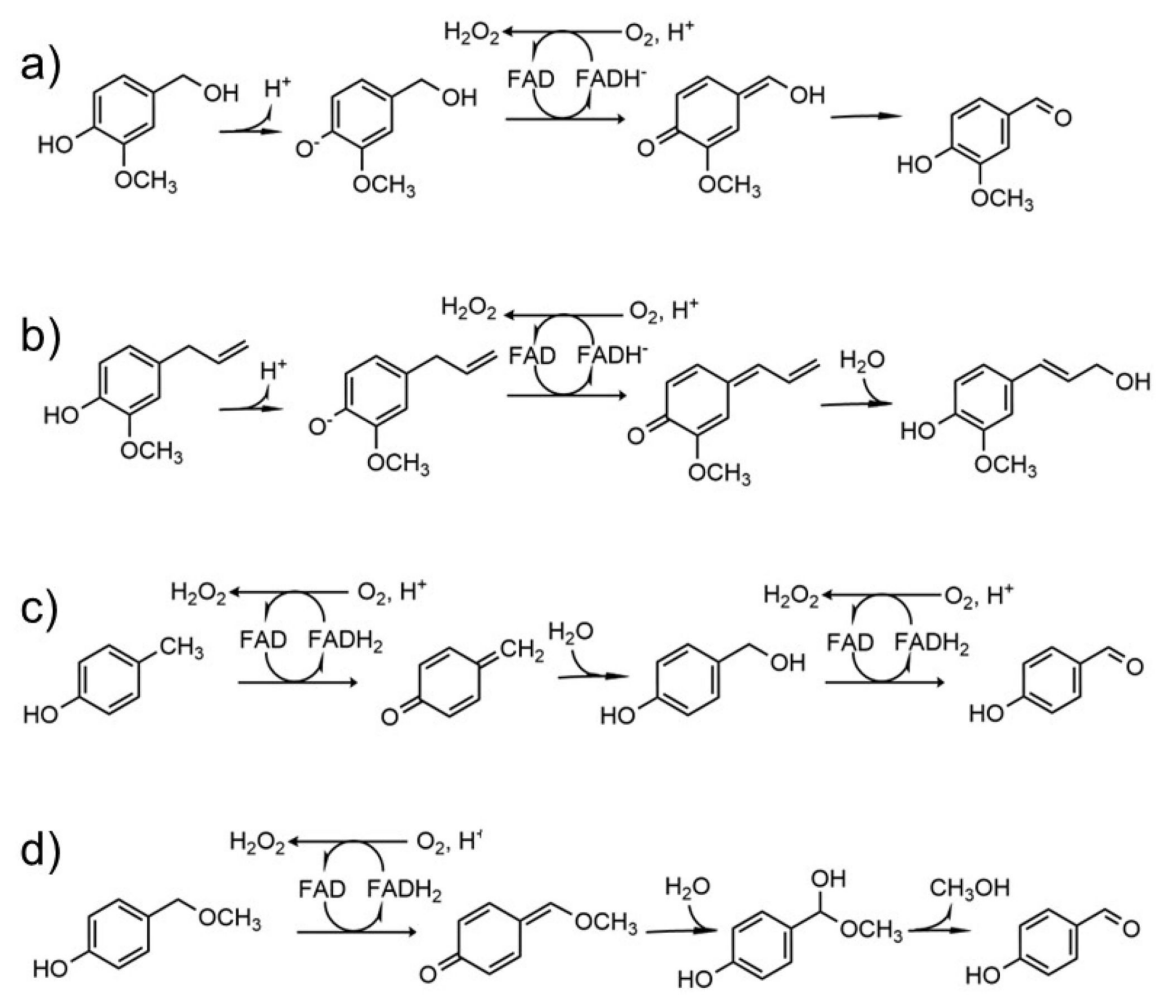
Some typical oxidations catalyzed by 4-phenol oxidizing enzymes. (**a**) Oxidation of vanillyl alcohol to vanillin; (**b**) hydroxylation of eugenol to coniferyl alcohol; (**c**) hydroxylation of *p*-cresol to 4-hydroxybenzylaldehyde; (**d**) oxidative demethylation of 4- (methoxymethyl)phenol to 4-hydroxybenzylaldehyde. The reactions are shown with reference to oxygen as electron acceptor.

Enzymes of the vanillyl alcohol oxidase/*p*-cresol methyl hydroxylase (VAO/PCMH) superfamily are found across all kingdoms where they perform a range of metabolic functions [1]. These flavoproteins share a conserved FAD-binding domain and a more variable substrate-binding domain that enables interactions with a range of substrates [2,3]. Phylogenetic analysis divides the VAO/PCMH family into 11 functionally different subgroups [4]. One of these, the 4-phenol oxidases/dehydrogenases, catalyzes the oxidation of *para*-substituted phenolic compounds. The reactions start with the transfer of a hydride anion from the Cα atom of the substrate’s *para*-substituent to the flavin that becomes two-electron reduced. This process yields a *para*-quinone methide intermediate that subsequently rearranges or reacts with water to yield the final product (Scheme 1). The reduced flavin must be re-oxidized to undergo another round of catalysis and, therefore, the reaction requires an electron acceptor [5–7]. A fundamental distinction is that the oxidases efficiently react with oxygen producing hydrogen peroxide whereas the dehydrogenases hardly react with oxygen and typically employ other proteins, such as cytochrome c, as acceptors [8]. Vanillyl alcohol oxidase (VAO), eugenol oxidase (EUGO), and 4-ethylphenol oxidase (EPO) are widely studied oxidases [5,9–11]; *p*-cresol methylhydroxylase (PCMH) and eugenol hydroxylase (EUGH) exemplify the dehydrogenases from the same subgroup [12–14].

The pervasive presence of the 4-phenol oxidizing enzymes across microorganisms and their metabolic diversity showcase these proteins as insightful systems to study how enzymes gain or lose the ability to utilize oxygen as electron acceptor during evolution [15–16]. Critical questions are: (i) Is the oxygen reactivity determined by a few selected residues or is it the composite outcome of many contributing factors? (ii) How do the oxygen-controlling residues and/or structural elements evolve along the evolutionary branches? (iii) How is the oxygen reactivity modulated by the protein matrix and the microenvironment of the prosthetic group? To address these issues, here we study two enzymes, from *Marinicaulis flavus* and *Novosphingobium* sp., respectively. Due to their amino acid sequences sharing characteristics with both dehydrogenases and oxidases, they were considered valuable models for exploring the evolution of oxygen reactivity in flavoenzymes. By combining several experimental approaches, we found that a specific site around the flavin is a pivotal determinant by controlling flavin accessibility through modulation of substrate binding and oxygen tunnel formation. The oxygen reactivity is thereby mostly determined by and evolves with the residues in direct contact with the reactive atoms of the flavin.

## Materials and methods

### Data collection and sequence analysis

A dataset of VAO/PCMH superfamily sequences was constructed by homology searches using blastp, restricting the searches to each domain of life. After curation, a dataset of 292 sequences (ranging from 464 to 654 amino acids length) was employed to construct a multiple sequence alignment in MAFFT V7 [17] and this was manually trimmed (292 sequences, 652 sites). A maximum likelihood phylogeny was inferred in RaxML v8.2.10 (HPC-PTHREADS module), with 500 rapid bootstrapping employing and automatic model calculation under gamma distribution (LG+F, α= 1.2). Transfer Bootstrap Expectation values were calculated with BOOSTER [18]. The phylogeny was also inferred in Mr. Bayes 3.2.6 for 1200000 generations until convergence (sd =0.15, α= 1.35). Figtree 1.4.2 was used to visualize and analyze the trees, and a consensus phylogeny was finally generated with TBE/PP (Transfer Bootstrap Expectation/Posterior Probability) values as statistical support at the nodes. Weblogo3 was employed to obtain logos for each analyzed clade [19].

#### Cloning, transformation, mutagenesis, and expression

Recombinant *Marinicaulis flavus* vanillyl alcohol dehydrogenase, *Rhodococcus jostii* RHA1 eugenol oxidase, and *Novosphingobium* sp. vanillyl alcohol oxidase were expressed using pBAD-His derived vectors (from GenScript) in *Escherichia coli* NEB10β RbCl-competent cells by arabinose induction. The plasmid transformation process involved incubating 2 μL of plasmid in 50 μL of cells on ice, followed by a brief 1-minute heat shock at 42 °C. Post-heat shock, the addition of 250 μL microliters of pre-warmed LB-SOC medium was followed by cell growth at 37 °C for an hour. Subsequently, 50 μL of the recovered cells were plated on LB-agar supplemented with 100 μg mL^−1^ ampicillin and incubated overnight at 37 °C. Then, a 5-milliliter pre-inoculum of LB-amp (100 μg mL^−1^) was cultured overnight at 37 °C and utilized to inoculate 2 L baffled flasks containing 500 mL of Terrific Broth medium supplemented with 100 μg mL^−1^ ampicillin. The flasks underwent incubation at 37 °C until an optical density at 600 nanometers (OD_600_) of 0.6 to 0.8 was obtained. Expression was induced with arabinose 0.02%. Following induction, the cells were left at 30 °C overnight and subsequently harvested *via* centrifugation (10,000 rpm, 30 min, 4 °C) before swift freezing in liquid nitrogen.

The QuikChange site-directed method was used for mutagenesis on recombinant *Marinicaulis flavus* vanillyl alcohol dehydrogenase, *Novosphingobium* sp. vanillyl alcohol oxidase, and *Rhodococcus jostii* RHA1 eugenol oxidase. The PCR final mix was composed of 10 μL of PfuUltra II Hotstart PCR Master Mix, 1 μL of primer forward and 1 μL of primer reverse (**Supplementary Information**), both at a starting concentration of 10 μM, 1 μL of plasmid DNA at 100 ng/μL, and MQ water up to 20 μL. This mixture was then subjected to PCR amplification, which resulted in the generation of a mutated gene. After the PCR reaction was completed, the samples were incubated with DpnI for 3 hours at 37 °C to digest the parental plasmid DNA. The samples were then used to transform new aliquots of NEB10β RbCl cells plated on LB-agar supplemented with 100 μg mL^−1^ ampicillin, by leaving them at 37 °C overnight. Plasmid isolation was performed to extract the mutated gene from the cells. Two colonies for each mutant were picked, pre-inoculated, and purified with QIAGEN’s QIAprep 8 Miniprep Kit. Finally, the presence of the mutations of interest was confirmed by Sanger sequencing.

#### Protein purification

Cell pellets were suspended in buffer A (100 mM potassium phosphate and 150 mM sodium chloride at pH 7.5) at a 3:1 volume-to-mass ratio, augmented with 1 mM phenyl methyl sulfonyl fluoride and 100 μM FAD. Cellular disruption was achieved *via* sonication (5-second on, 5-second off cycle at 70% amplitude for a cumulative duration of 10 minutes) followed by centrifugation at 10,000 rpm for 1 hour. The resultant supernatant was then loaded onto a nickel affinity chromatography column housing 2 mL of Ni sepharose resin, which had been previously equilibrated with buffer A. After a round of washing with buffer B (50 mM potassium phosphate, 150 mM sodium chloride, and 20 mM imidazole), proteins were eluted (over 2 column volumes) using buffer C (50 mM potassium phosphate, 150 mM sodium chloride, and 500 mM imidazole at pH 7.5). To eliminate the high imidazole content from the samples, a PD-10 desalting column (Cytiva) was employed. This column was initially prepared with buffer A, and the purified protein samples were subsequently eluted using the same buffer.

#### UV-visible spectra

UV-visible spectral analyses were performed using an Agilent 8453 diode array spectrophotometer. Spectral changes were observed by mixing 30 μM enzyme with 45 μM vanillyl alcohol or 1 mM of dithionite, monitored for 30 minutes. Similarly, spectral changes were measured by mixing 17 μM wild-type or P151L VADs with 4-(methoxy)methylphenol at concentrations of 17 μM, 34 μM, and 68 μM, monitored for 5 minutes.

#### Differential scanning fluorimetry

The apparent melting temperatures (T_m_) were measured with a differential scanning fluorimetry assay employing a Tycho N6 instrument from Nanotemper. Capillaries were prepared with a final volume of 10 μL, comprising 9 μL of buffer (10 mM Tris-HCl buffer at pH 7.5 at 4 °C) alongside 1 μL of gel-filtered protein, maintained at a concentration of 0.7 mg/mL. The differential scanning fluorimetry assay involved recording fluorescence measurements across a gradual temperature range, progressively escalating from 35 °C to 95 °C.

#### Steady state kinetics

Steady state kinetics were performed at increasing the concentration of substrates (final 1% v/v DMSO) in buffer A. These solutions were blended with 0.5-4 μM of protein in a total volume of 100 μL. Dehydrogenase activity was detected by monitoring the reduction of 2,6-dichlorophenolindophenol (DCPIP, ε_600nm_=19,000 M^−1^cm^−1^) or bovine heart cytochrome c (ε_550nm_=21,000 M^−1^cm^−1^; from Merck). Oxidase activity was detected by monitoring product formation (vanillin, ε_340nm_=14,000 M^−1^cm^−1^: 4-hydroxybenzaldehyde, ε_329nm_=10,600 M^−1^cm^−1^; coniferyl alcohol, ε_296nm_=6,800 M^−1^cm^−1^) using a CARY 100 (Agilent) instrument. The rates observed at varying substrate concentrations were analyzed using GraphPad Prism and fitted to the Michaelis-Menten equation, providing K_M_ (μM) and k_cat_ (s^−1^) values. Each experiment was performed in triplicate.

#### Stopped-flow kinetics on VAD

The stopped-flow measurements were performed using an SX20 stopped-flow spectrophotometer equipped with a photodiode array detector (Applied Photophysics, Surrey, UK). Enzyme solutions were made anaerobic by flushing the vial with nitrogen for 15 min. Moreover, 5 mM of glucose and 0.3 μM of glucose oxidase (*Aspergillus niger*, type VII, Sigma-Aldrich) were added to the solution to remove the leftover oxygen. For estimation of rate constants, a diode-array absorbance detector was used. For the reductive half-reaction, the measurements were performed using five different vanillyl-alcohol concentrations. For the oxidative half reaction, proteins were anaerobically reduced with a 1.5-fold excess of vanillyl alcohol or dithionite. Re-oxidation of reduced enzymes was measured after mixing with two or four different oxygen concentrations. Re-oxidation of dithionite-reduced wild-type protein and vanillyl alcohol-reduced P151L mutant was also measured with an excess of vanillin (1 mM) in order to see if the product inhibits oxidase activity. For enzyme-monitored turnover experiments, air-saturated proteins were mixed with vanillyl alcohol in the stopped-flow instrument after which the redox state of the flavin cofactor was recorded through diode-array absorbance detector. All data were analyzed using the Pro-Data software (Applied Photophysics) and GraphPad Prism.

#### Redox potential determination

The redox potential of wild-type and P151L VADs was determined using the xanthine/xanthine oxidase methodology [20]. The reaction was performed in 50 mM potassium phosphate buffer (pH 7.5) and 150 mM NaCl at 25 °C using a 1 mL quartz cuvette. The reaction mixture consisted of 2.0 μM benzyl viologen, 5.0 μg/mL catalase, 300 μM xanthine, 0.3 nM xanthine oxidase, 0.6 μM glucose oxidase, and 50 mM glucose. An anaerobic condition was created by flushing the cuvette with argon for 15 min after which the glucose/glucose oxidase system assured fully anoxic conditions. Xanthine was added to initiate the redox titration. Spectra were recorded for 1 h. Thionine acetate (E_0_= 60 mV) was found to be a suitable dye for determining the redox potential of VAD while for P151L methylene blue (E_0_= 11 mV) was used as reference dye. The E_m_ values were calculated by applying the Nerst equation.

#### Protein crystallization, X-ray data collection, and structure determination

After cleavage with SUMO protease, all purified proteins were subjected to gel filtration purification, utilizing a superdex 200 10/300 column (Cytiva) connected to an ÄKTA Pure system. Monitoring the elution of proteins during this process involved the use of two wavelengths, 280 and 450 nanometers (nm). Fractions from the elution peaks were pooled and concentrated to 13 mg/mL in a 10 mM Tris-HCl buffer at pH 7.5 at 4 °C. Crystallization was performed using the vapor-diffusion sitting-drop technique at 20 °C by mixing equal volumes of protein and precipitant solutions that varied depending on the protein variant as follows:

- Wild-type VAD: 0.2 M ammonium chloride and 20% w/v PEG 3350;
- P151L: 0.09M NPS (0.3 M sodium nitrate; 0.3 M sodium phosphate dibasic; 0.3 M ammonium sulfate), 0.1 M buffer system 1 pH 6.5 (imidazole; MES monohydrate), and 50% v/v precipitant mix 4 (25% v/v MPD; 25% v/v PEG 1000 and 25% w/v PEG 3350);
- P151N and P151T: 0.2 M NaCl and 20% w/v PEG 3350;
- P151I: 0.2 M potassium iodide with 20% w/v PEG 3350;
- P151G: 0.1 M carboxylic acids (0.2 M sodium formate, 0.2 M ammonium acetate, 0.2 M sodium citrate tribasic dihydrate, 0.2 M sodium potassium tartrate tetrahydrate, 0.2 M sodium oxamate); 0.1 M buffer system 1 pH 6.5 (imidazole and MES monohydrate) and 50% v/v precipitant mix 4 (25% v/v MPD, 25% PEG 1000, 25% w/v PEG 3350);
- Wild-type nsVAO:0.1 M amino acids (0.2 M L-Na-glutamate, 0.2 M alanine (racemic), 0.2 M glycine, 0.2 M lysine HCl (racemic), and 0.2 M serine (racemic)), 0.1 M buffer system 1 at pH 6.5 (imidazole and MES monohydrate (acid)), and 50% v/v precipitant mix 2 (40% v/v ethylene glycol and 20% w/v PEG 8000).
- T181D nsVAO:0.1 M MES at pH 6.5, 15% PEG 20000, and 25% glycerol.

P151L VAD-eugenol, wild type nsVAO-vanillyl alcohol and T181D nsVAO-vanillin complexes were prepared by soaking crystals (2 minutes) in solutions consisting of the same cryoprotectant solution for each variant with 1 mM of eugenol and vanillyl alcohol, respectively.

Crystallographic data were collected at the Massif3 beamline of the European Synchrotron Radiation Facility (ESRF) in Grenoble, France. All crystals diffracted with a resolution between 1.6 and 2.5 Å. Diffraction images were processed with XDS and Aimless from the CCP4i software suite [21–22]. Molecular replacement was used to solve the crystal structures, with the coordinates of from AlphaFold3 as the search models. Atomic models were refined with Refmac5 and Coot [23–24] and analyzed with Caver [25]. Figures were created with ChimeraX [26] and Pymol (https://www.pymol.org). Crystallographic statistics are listed in Table S1.

#### Molecular dynamics simulations and analysis of O_2_ diffusion

The PDB files of the crystal structures of wild-type and P151L VADs were initially preprocessed with the pdb4AMBER and reduce utilities (both from AmberTools21) [27] to add missing hydrogen atoms and to settle the correct tautomers and rotamers of histidines, asparagines and glutamines. PropKa (version 3.1) [28] was used to determine likely protonation states of titratable residues at pH 7. Atomic point charges for non-standard molecules and residues were computed using Gaussian16. Molecular dynamics (MD) simulations were performed using the AMBER20 software suite, specifically with the GPU-accelerated Particle Mesh Ewald Molecular Dynamics (Pmemd) engine for the production stage [29]. The force fields used included ff14SB for standard protein residues [30]. Generalized Amber Forcefield (gaff) parameterizations [31] for non-standard residues such as FAD and quinone methide intermediate (QMI), parameters by Wang *et al*. for dioxygen [32], Joung-Cheatham parameters for Na^+^ and Cl^–^ counterions (ionic strength 0.1 M), and TIP3P for water modeling [33]. In particular, parameters for FAD had to be slightly modified, and the covalent bond between His387 and FAD and associated bonded parameters were modeled by borrowing the chemically closest parameters available in the gaff forcefield [32]. Solvent equilibration, heating from 25 K to 300 K, and preproduction were carried out over a total of 4.069 ns, switching from the NVT to the NpT ensembles. Regarding production, six independent MD replicas were run, for each variant, covering 500 ns of simulation time in the NpT ensemble at standard conditions. The initial conditions for all simulations were set with all O_2_ molecules intentionally placed outside the VAD variant of interest. A 100 x O_2_ concentration excess was used.

The diffusion of O_2_ molecules inside the protein was analyzed using the MDpocket Package with the following optimized parameters for the detection of very small channels and pockets (-m 2.8, -M 6.0, -i 3, -n 2) [34]. Prior to analyzing the cavities in each protomer, two separate alignments of the MD trajectories were conducted, using the backbone heavy atoms of residues within 5 Å of the flavin ring. Pockets and channels present for at least 80% of the simulation time were considered in the analysis.

## Results

### Sequence analysis and phylogenesis of the VAO/PCMH family

**Figure 1:**
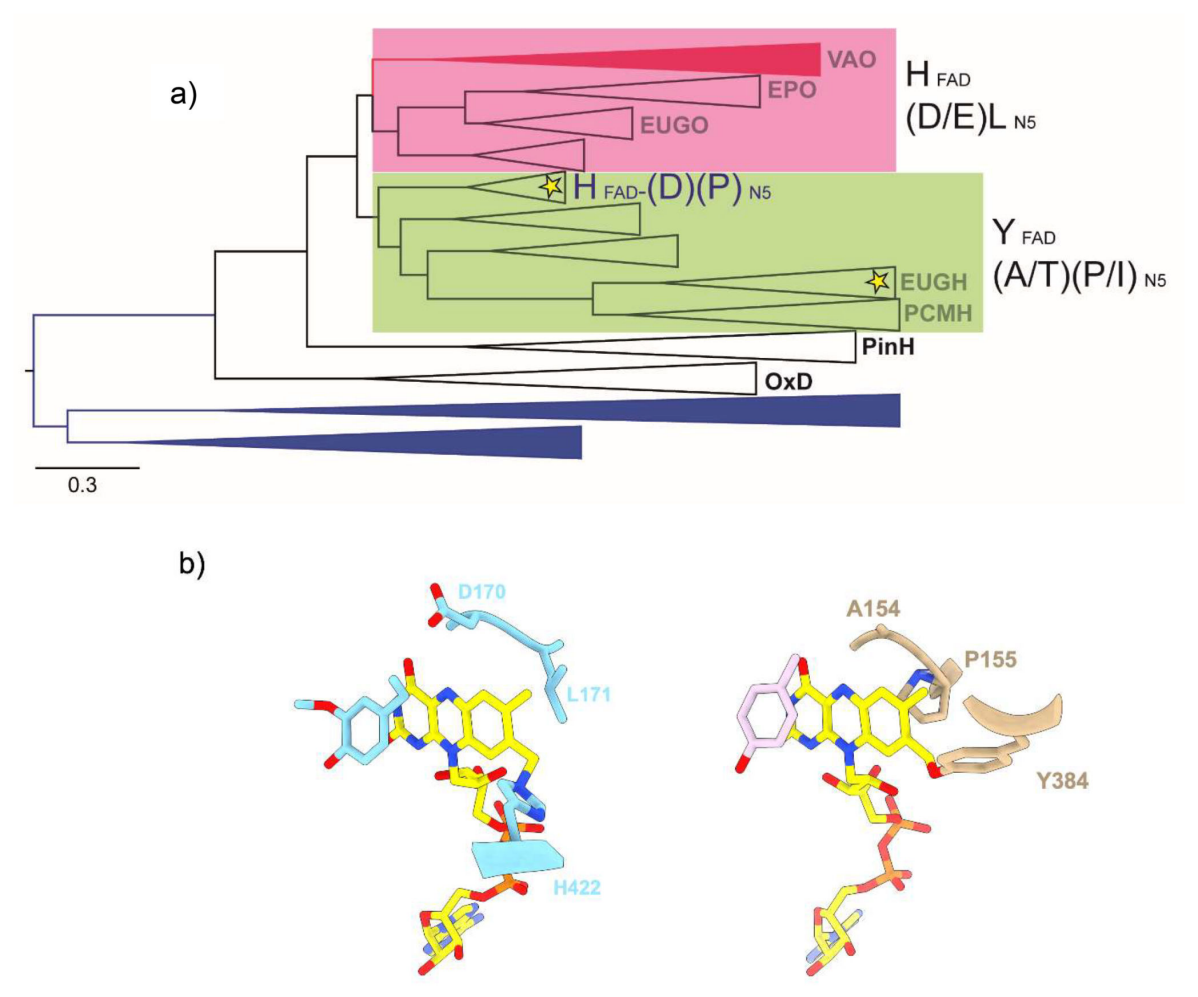
The VAO/PCMH enzymes acting on 4-phenol substrates. **(a)** Condensed phylogeny. Color of branches indicate the taxonomic distribution: Archaea (blue), Bacteria (black) and Eukarya (red). The clades are names after their experimentally characterized, archetypal enzymes. OxD and PinH identify oxidative decarboxylases and pinoresinol hydroxylases, respectively; they are not part of this study. Oxidases are highlighted in pink, dehydrogenases in green. The locations of the enzymes identified in this work, vanillyl-alcohol dehydrogenase from *Marinicaulis flavus* and vanillyl-alcohol oxidase from *Novosphingobium* sp., are marked by yellow stars. On the right, the identity of the signature residues involved in the covalent binding or in contact with the N5 site of FAD are shown (Table 1). Please refer to Figure S1 for the fully annotated tree. The scale bar indicates substitutions per site. **(b)** Active site residues in VAO from *Penicillium simplicissimum* (right; PDB:2VAO) and PCMH from *Pseudomonas putida* (left, PDB:1DIQ).

We constructed a robust dataset for the 4-phenol oxidases mining the three domains of life and inferred a robust phylogeny by maximum likelihood and Bayesian inference methods. The topology of the consensus tree obtained suggests an early divergence between two major clades represented by known oxidases (VAO, EPO, EUGO) and dehydrogenases (EUGH, PCMH) (Figure 1a, Figure S1). The degree of conservation analysis of these clades highlighted some key amino acids occupying critical sites around the prosthetic group. This was further confirmed by the structural analysis of well-known representatives (Figure 1b). The oxidases typically contain a conserved pair formed by a leucine and an acidic amino acid (Asp or Glu) in contact with the N5 atom of the flavin [35]. In the dehydrogenases, these two sites are systematically replaced by a proline or isoleucine and a small side-chain amino acid (*e.g*., Ala, Thr), respectively (Figure 1b, Table 1). In addition, the oxidases feature a histidine involved in a 8α-N3-histidyl-FAD covalent linkage whereas the dehydrogenases lack this histidine and rather comprise a tyrosine in a non-homologous position, forming a 8α-O-tyrosyl-FAD bond [14]. Given this consistent pattern, we reasoned that these residues, might have a role in the enhancement or suppression of the oxygen reactivity and thus being a signature of the electron acceptor preference.

Starting from this hypothesis and with the goal of addressing the evolution of oxygen reactivity in this group of flavoproteins, we noticed a clade featuring some distinctive properties as it exhibits the flavin-interacting aspartate and flavin-linking histidine typical of the oxidases while retaining the N5-interacting proline of the dehydrogenases (Figure 1a). This clade shares a remote ancestry with the PCMHs and EUGHs and the function of its members was generally unknown with no experimental characterization reported. Among the proteins of this unexplored clade, we chose the enzyme from *Marinicaulis flavus* for experimental evaluation (Table 1). The sequence and phylogenetic analysis additionally outlined a peculiar enzyme from *Novosphingobium* sp. that belongs to the EUGH clade and distinguishes itself for lacking a flavin-linking residue and the presence of a valine in the N5-interacting site (Figure 1a; Table 1). Thus, it represents an unusual family member that naturally binds the flavin non-covalently. Based on these features, we identified the enzymes from *Marinicaulis flavus* and *Novosphingobium* sp. as insightful candidates for biochemical and structural studies addressing the evolution and mechanisms underlying the dehydrogenase *vs* oxidase distinction (Table S2).

### VAD is a thermostable dehydrogenase most active on vanillyl alcohol and eugenol

**Figure 2:**
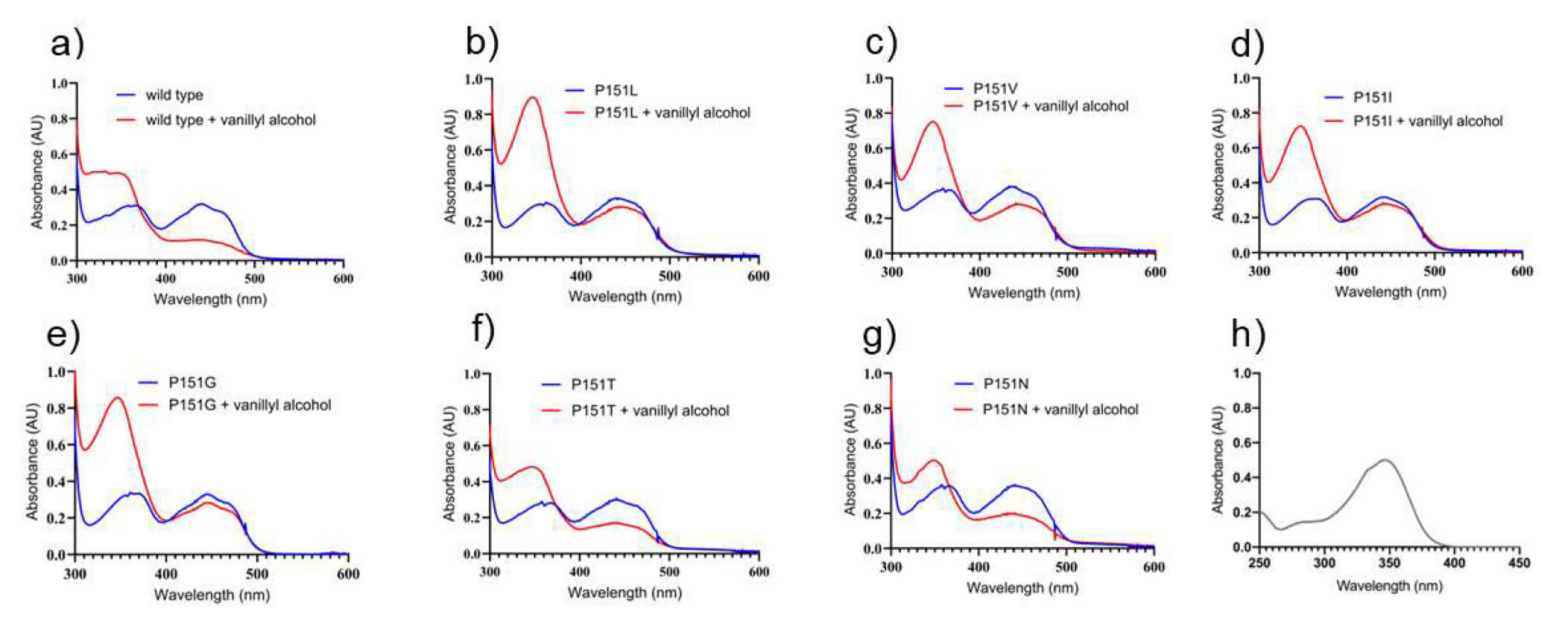
Spectral changes observed upon aerobic mixing of VAD with vanillyl alcohol. (**a**) Wild-type VAD; (**b**) P151L; (**c**) P151V; (**d**) P151I; (**e**) P151G; (**f**) P151T; (**g**) P151N. Protein and vanillyl alcohol concentrations are 30 μM and 45 μM, respectively. The spectra before substrate addition and after 30-minute incubation are in blue and red, respectively. (**h**) For reference, the absorbance spectrum of 30 μM vanillin is shown (ε_340nm_=14000 M^−1^·cm^−1^). Wild-type, P151N, and P151T VADs do not re-oxidize even after 30 minutes of exposure to air.

VAD from *Marinicaulis flavus* can be expressed in *Escherichia coli* with high yields. It is soluble, thermally stable (T_m_=65.5 °C), displays the characteristic flavoprotein absorbance spectrum with absorption maxima at 360 nm and 440 nm (Figure 2a, blue line) and a high redox potential, as often found in covalent flavoproteins (E_m_=+82±2 mV, Figure S3). It is active towards vanillyl alcohol, 4-hydroxybenzyl alcohol, eugenol, and 4- (methoxymethyl)phenol but inactive on *p*-cresol (Scheme 1, Table 2). For all substrates, the enzyme shows activity only when assayed using 2,6-dichlorophenolindophenol, a redox dye, as electron acceptor. The lack of activity with bovine heart cytochrome c suggests that the enzyme might be specifically active only with an endogenous (e.g. from *Marinicaulis flavus)* cytochrome c as observed for other flavoproteins [36–37]. Spectrophotometric measurements of the enzyme aerobically incubated with a 1.5-fold excess of vanillyl alcohol demonstrated that the enzyme remains reduced even if exposed for hours to air (Figure 2a, red line). The absorbance spectrum suggests that vanillin (peak at 340 nm; Figure 2) is produced, though the presence of the quinone methide intermediate (peak at 360 nm) remaining bound to a fraction of the protein molecules cannot be ruled out. These features are consistent with molecular oxygen being an inefficient oxidant for the enzyme that is therefore identified as a vanillyl-alcohol dehydrogenase active on the typical substrates of the 4-phenol oxidizing subgroup [2].

### A single amino acid substitution converts VAD into an oxidase

Having identified VAD from *Marinicaulis flavus* as a dehydrogenase, we investigated whether the dehydrogenase-characterizing proline around the flavin N5 locus has a role in suppressing the oxygen reactivity (Table 1). We first prepared and studied the P151L mutant, intended to mimic the N5 environment typical of the oxidases. The result was clear-cut: P151L-VAD can use dioxygen as an effective substrate for flavin re-oxidation. The mutant displays similar or even higher activities as oxidase when compared to the wild-type enzyme (e.g. on 4- (methoxymethyl)phenol; Table 2, Table S3). Consistently, vanillyl alcohol-incubated P151L-VAD is re-oxidized by oxygen with the build-up of vanillin in large amounts and no accumulation of the enzyme’s reduced form (Figure 2b). Therefore, P151L-VAD can be regarded as a competent oxidase.

To further elucidate the role of the N5-interacting position, additional amino acid replacements were studied. Pro151 was substituted with isoleucine and valine to mimic the N5 environment of EUGHs (Table 1). We found that P151V and P151I can operate with oxygen although they are 10- to 40-fold less efficient compared to using 2,6-dichlorophenolindophenol as co-substrate (Figure 2c-d, Table 2). We further studied the P151G mutation to assess the effect of removing *en bloc* the side chain, and P151N and P151T to test the introduction of hydrogen-bonding groups. The P151G mutation was found to elicit the same oxidase-activating effect of P151L whereas P151T and P151N conserve the properties of a dehydrogenase (Table 2). Spectrophotometric measurements confirmed that the vanillyl alcohol-reduced P151N and P151T proteins are hardly re-oxidized by molecular oxygen though they retain dehydrogenase activity while P151G can effectively turn over using oxygen as co-substrate and the product is formed (Figure 2e-g). Collectively, these mutagenesis experiments demonstrate that even single amino acid substitutions near the reactive N5 atom of the flavin can convert a dehydrogenase into an oxidase, although additional sites beyond position 151 likely further regulate its reaction with oxygen.

### The poor reactivity with oxygen is not an intrinsic property of the VAD-bound flavin

**Figure 3:**
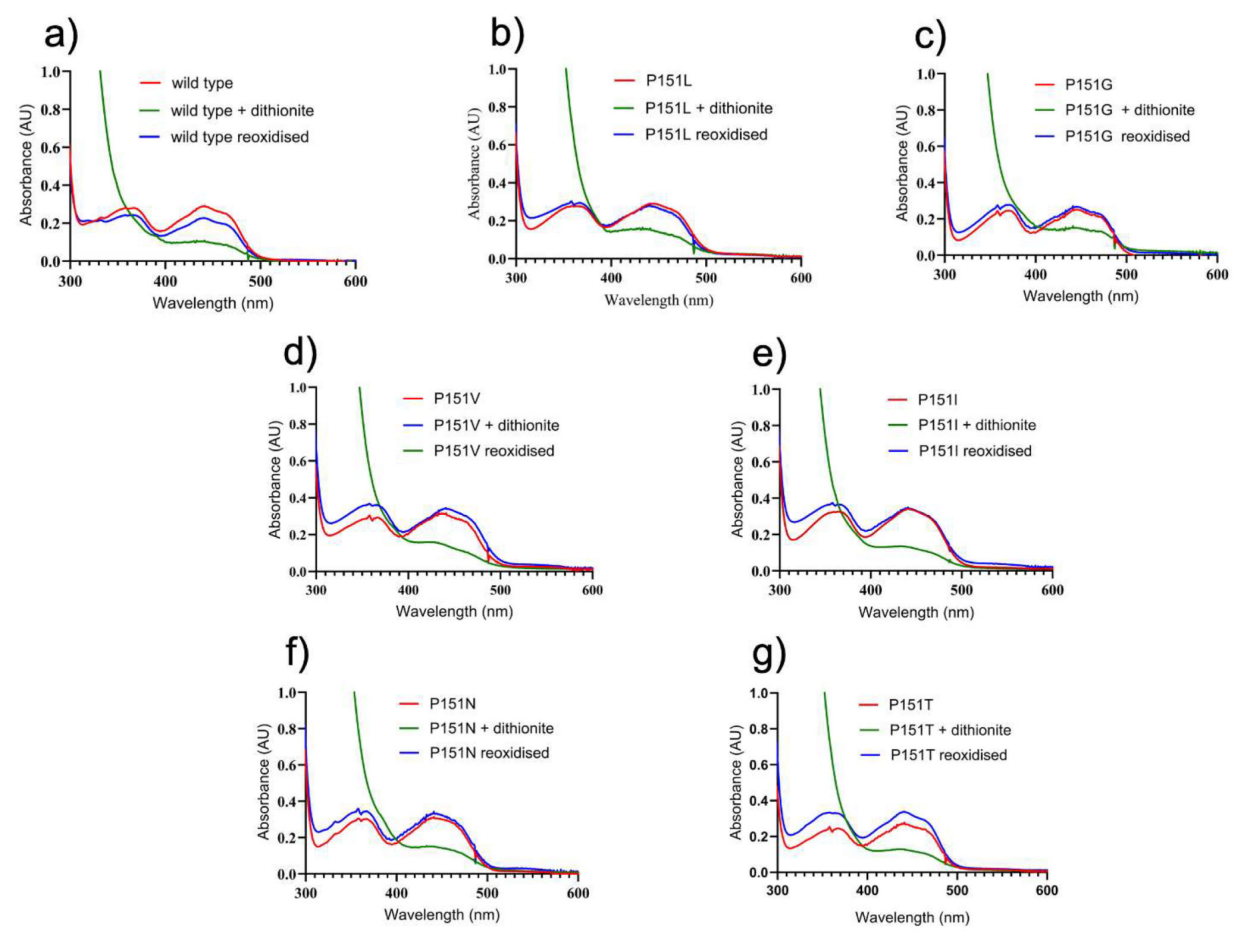
Spectral changes observed upon aerobic mixing of VAD with dithionite. (**a**) Wild-type; (**b**) P151L; (**c**) P151G; (**d**) P151V; (**e**) (P151I; (**f**) P151N; (**g**) P151T. Protein and dithionite concentrations are 30 μM and 1.5 mM, respectively. The spectra measured before and immediately after the addition of dithionite, and after 10-minute incubation are in red, green, and blue, respectively. In all cases, the proteins are re-oxidized after 10 minutes.

The microenvironment around the cofactor can, in principle, alter the inherent chemical properties of the flavin, either suppressing or boosting its oxygen reactivity when bound to the hosting protein [15,38]. Also, binding of ligands next to a protein-bound flavin can affect its ability to react with oxygen [39]. To investigate the role of the microenvironment in VAD in its ability to utilize molecular oxygen as electron acceptor, we used dithionite as a substitute of the substrate to reduce the enzyme. The experiment yielded highly informative results: the dithionite-reduced wild-type and mutant VADs are both re-oxidized by oxygen (Figure 3). This finding indicates that the VAD-bound flavin is not inherently inert towards oxygen. Instead, its reactivity is influenced by the type of reductant used, whether it is the natural substrate or a non-specific reagent like dithionite. This hints to a role of ligand binding in the active site, affecting oxygen reactivity.

**Figure 4:**
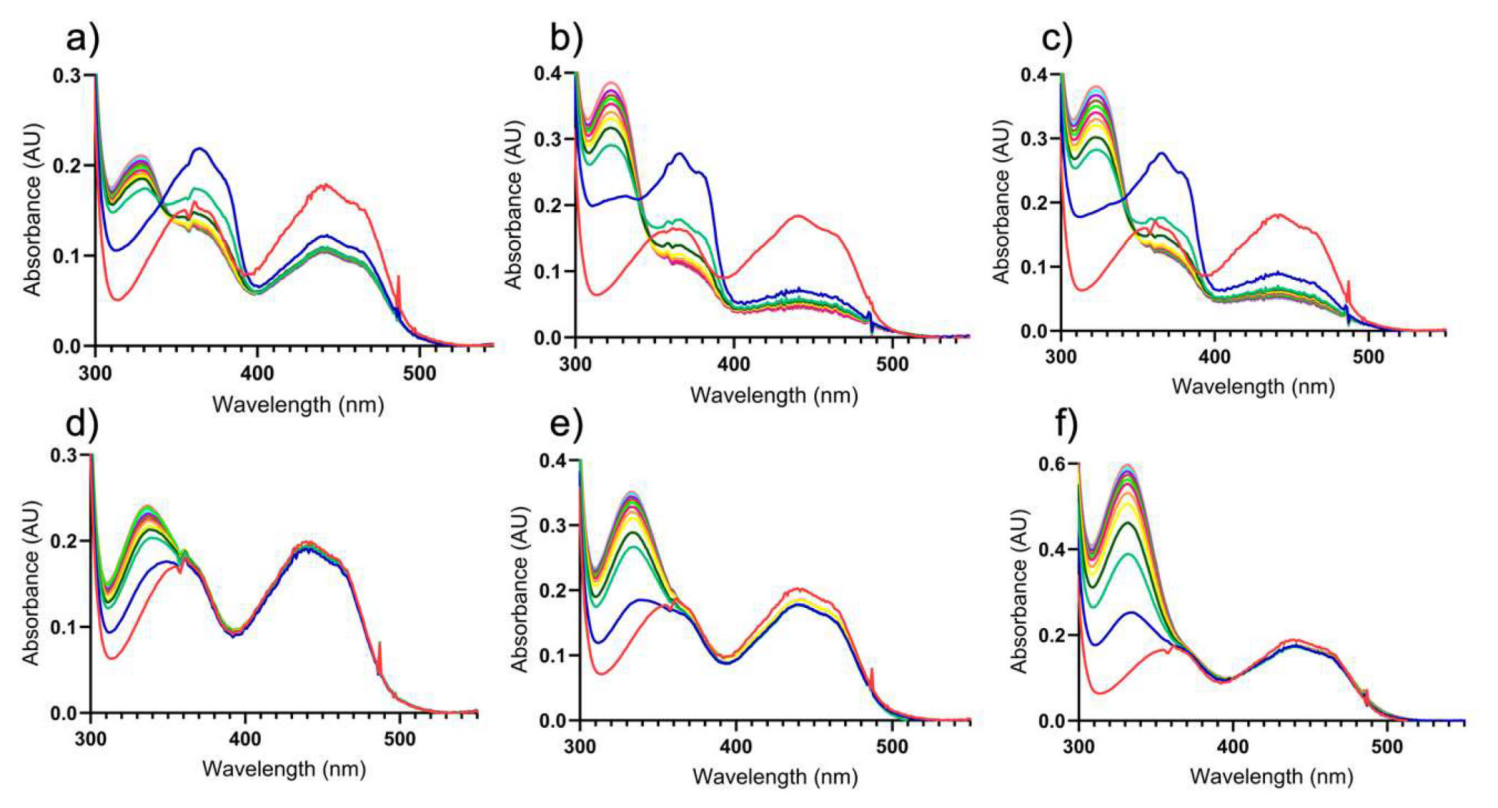
Spectral changes observed upon aerobic mixing VAD with 4-(methoxy)methylphenol. **(a-c)** 17 μM wild-type VAD mixed with (**a**) 17 μM, (**b**) 34 μM, and (**c**) 68 μM 4-(methoxymethyl)phenol. (**d-f**) 17 μM P151L VAD mixed with (**d**) 17 μM, (**e**) 34 μM, (**f**) 68 μM 4-(methoxymethyl)phenol. The spectra represent the enzymes before (red) and immediately after the addition of 4-(methoxymethyl)phenol (blue) followed by incubations for 30 seconds (green), 1 minute (dark green), 1.50 minute (yellow), 2 minutes (orange), 2.50 minutes (purple), 3 minutes (light green), 3.50 minutes (brown), 4 minutes (violet), 4.50 minutes (light blue) and 5 minutes (pink). The quinone methide intermediate initially formed upon 4-(methoxy)methylphenol oxidation absorbs at 364 nm (ε=46000 M^−1^·cm^−1^) and is clearly visible in the early incubation times of the wild-type protein. The intermediate is then attacked by a water to generate 4-hydroxybenzaldehyde that has an absorbance peak at 329 nm (ε=10600 M^−1^·cm^−1^; Scheme 1d).

Considering the above-described results, we next aimed to understand how substrate selectively suppresses the oxygen reactivity in the wild-type VAD. To this end, we first focused on 4-(methoxy)methylphenol, a slow substrate (Scheme 1d, Table 2). Its reaction can be easily monitored by absorbance spectroscopy as both its quinone methide intermediate and aldehyde product exhibit distinct absorption peaks in the UV-visible range (Figure 4). Upon aerobic incubation of VAD with increasing concentrations of this substrate, we observed the generation of the reduced enzyme and the quinone methide intermediate, whose spectroscopic signature is clearly detectable in the initial spectra (Figure 4a-c). However, the intermediate accumulates only transiently, as it hydrolyzes to the aldehyde product while the enzyme remains in the reduced state. These findings suggest that the suppression of oxygen reactivity in wild-type VAD is not due to the formation of a stable, oxygen-inert complex between the reduced enzyme and the quinone methide. Instead, the intermediate to a large extent decays, yet the flavin remains inefficiently oxidized by oxygen, unlike the P151L oxidase mutant (Figure 4d-f).

### The P151L mutation affects both flavin reduction and oxidation in VAD

**Figure 5:**
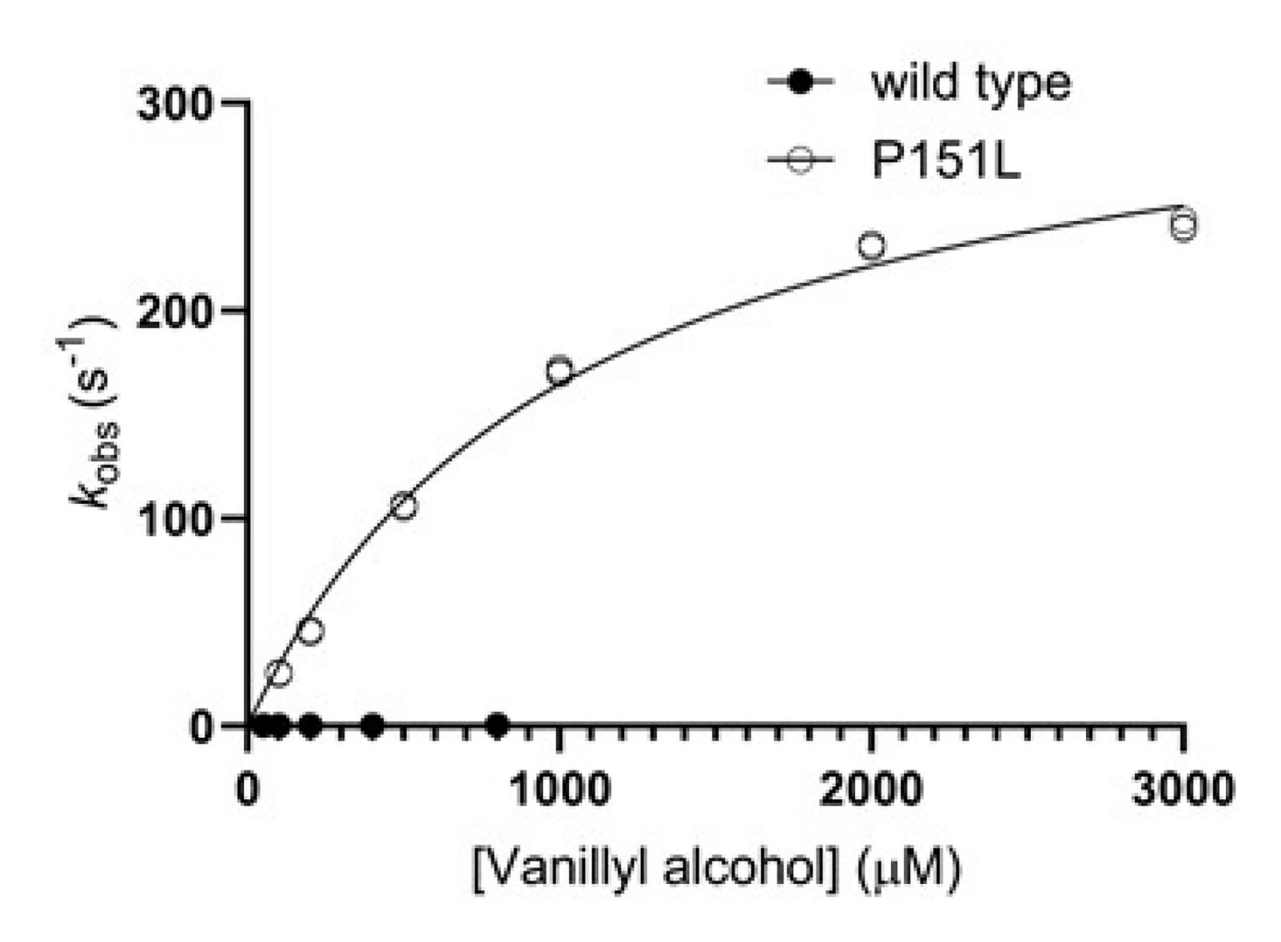
The reductive half-reaction in VAD. Anaerobic reduction rates of wild-type (5 μM, dark circles) and P151L (5 μM, light circles) VADs at different vanillyl alcohol concentrations (50-800 μM for the wild-type enzyme; 100-3000 μM for P151L). n = 3 independent experiments, individually plotted as dots. See also Figure S7.

The experiments described above establish that the oxygen-reactivity of VAD is modulated by the substrate (or product) and this effect depends on the residue in direct contact with the flavin N5 atom. To further investigate the kinetics of flavin reduction and oxidation underlying these phenomena, we used stopped-flow methods with the wild-type (dehydrogenase) and P151L (oxidase) enzymes as reference systems [40]. We found that wild-type VAD is anaerobically reduced by vanillyl alcohol with a reduction rate constant (*k*_red_) of 0.50 s^-1^ (reductive half-reaction; Table 3, Figure 5). The formation of a strong absorption peak at 350 nm represents vanillin generation possibly mixed with some quinone methide intermediate withheld by the enzyme (Figure S7a). P151L is even more rapidly reduced with a *k*_red_ value of 339 s^-1^, which is 600-fold faster than in the wild-type enzyme (Table 3, Figure 5, Figure S7b). Notably, within less than a second, a rapid shift in absorbance from 355 to 350 nm indicates the release of the product from the enzyme’s active site. The striking difference between the flavin reduction kinetics between wild type and P151L is not due to the redox potential, because similar redox potentials were measured for both VAD variants (Figure S3). Interestingly, we also found that the P151L enzyme binds the substrate with 40-fold lower affinity than the wild type. These data demonstrate that mutating the flavin-interacting Pro151 residue considerably affects the reductive reaction. While flavin reduction in P151L is much faster, the mutant binds the substrate less avidly than the wild-type protein.

**Figure 6:**
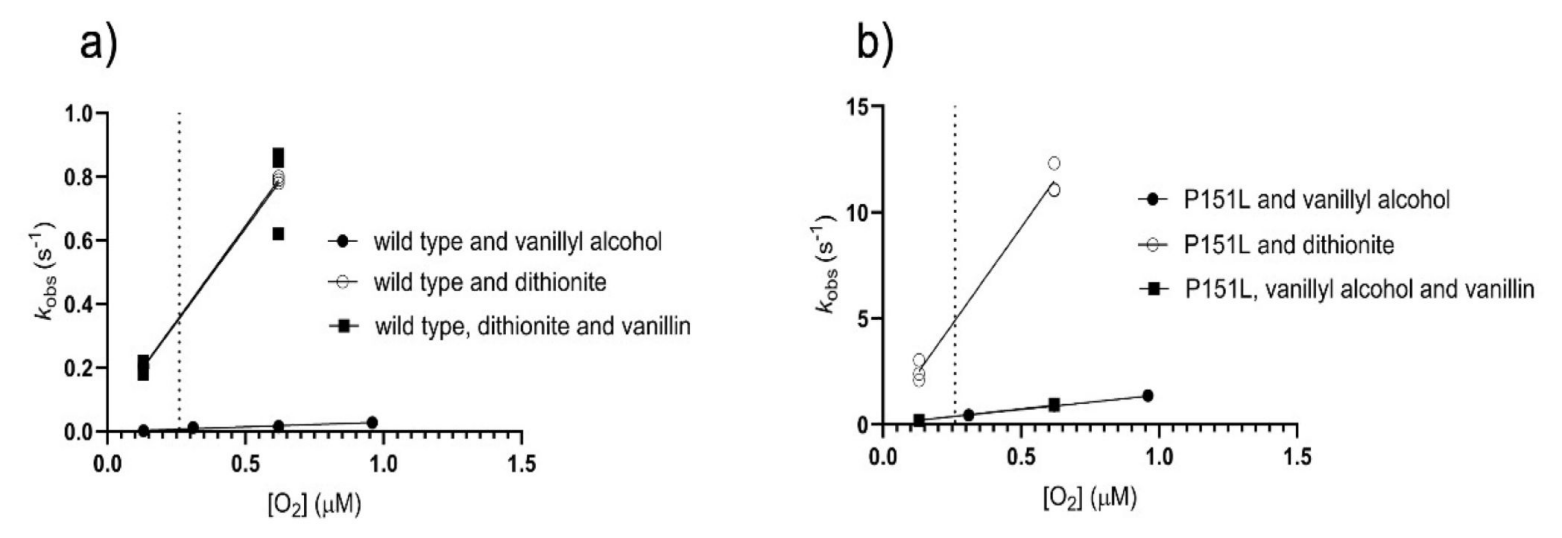
The oxidative half-reaction in VAD. (**a**) Wild type VAD (5-20 μM) reduced with 1–1.5 equivalents of vanillyl alcohol (dark circles), dithionite (light circles), and dithionite in presence of 1 mM of vanillin (dark squares) were mixed with different dioxygen concentrations. The dotted line indicates the atmospheric oxygen concentration. (**b**) P151L (5-10 μM) reduced with 1–1.5 equivalents of vanillyl alcohol (dark circles), dithionite (light circles), and vanillyl alcohol in presence of 1 mM of vanillin (dark squares) were mixed with different dioxygen concentrations. n = 3 independent experiments, individually plotted as dots. Please note the different scales of the vertical axes. The kinetics constants are listed in Table 4.

In agreement with the spectroscopic data (Figures 2a-3a), the stopped-flow experiments confirm that the reactivity of wild-type VAD with oxygen depends on the reductant used. Specifically, the re-oxidation of the dithionite-reduced enzyme is 52-fold faster than that of the substrate-reduced protein (*k*_ox_ of 14000 *vs* 27 M^-1^ s^-1^; Figure 6a, Table 4). Notably, the addition of an excess of the reaction product has no effect on the process. Furthermore, the stopped-flow data showcase the drastic effect imparted by the P151L mutation: the re-oxidation of the substrate-reduced mutant is 55-fold faster than that of the wild-type enzyme (*k*_ox_ of 1500 *vs* 27 M^-1^ s^-1^; Figure 6b, Table 4, Figure S8). This rate increases further when dithionite is used as the reducing agent, whereas the presence of vanillin has no effect. Together, these data reinforce the concept that the substrate, rather than the product, inhibits the reaction of the wild-type VAD with oxygen. They suggest a model in which quinone-methide retention or substrate binding to the reduced enzyme protects it from oxygen-mediated re-oxidation. The Pro151 mutation mitigates this effect, possibly by reducing the enzyme’s affinity for the substrate, as suggested by the 40-fold lower K_d_ value for substrate binding to the oxidized wild-type enzyme compared to P151L (Table 3).

### Pro151 mutations affect the local environment and accessibility of the flavin

**Figure 7:**
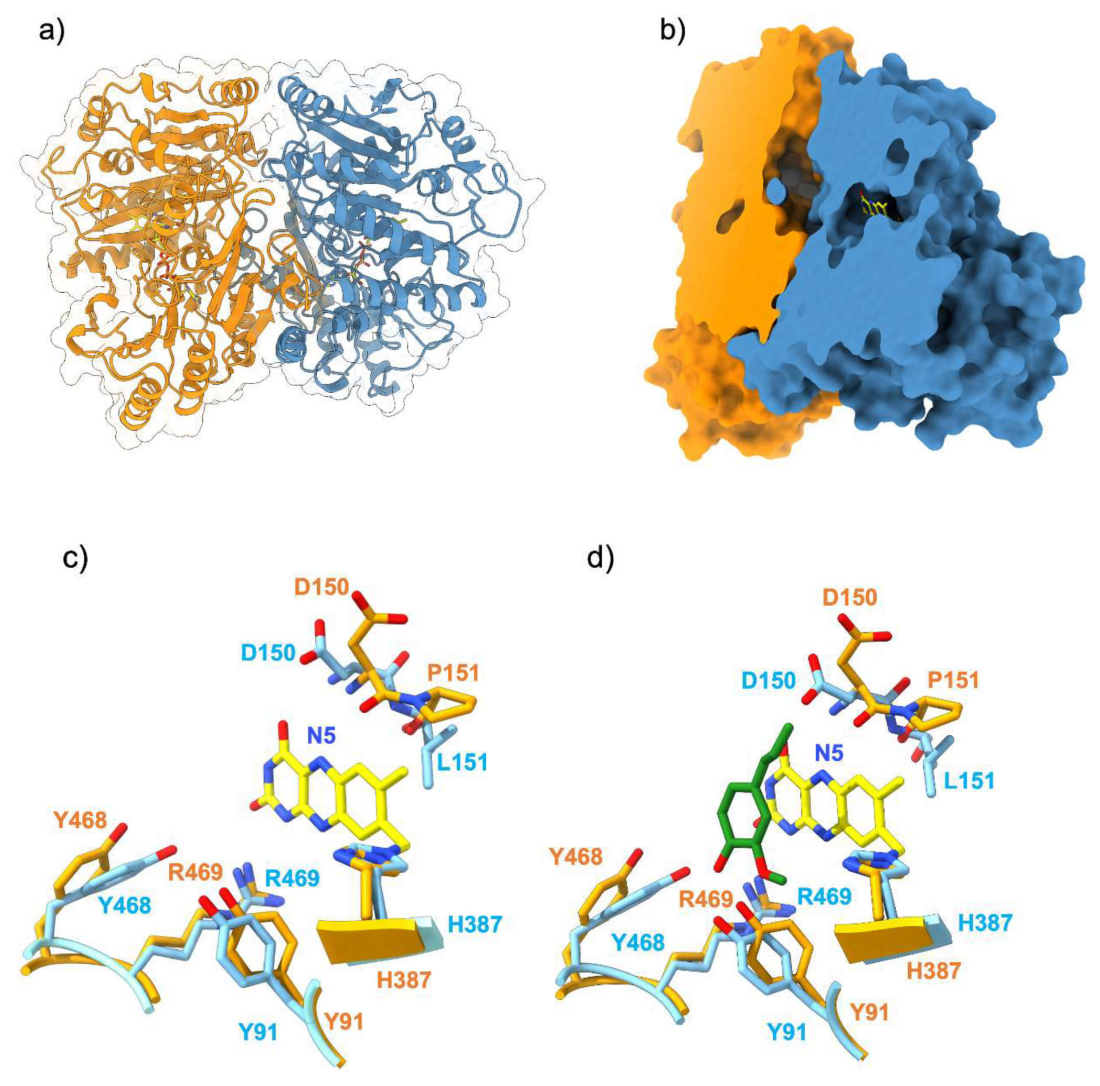
The three-dimensional structure of VAD. (**a**) Ribbon representation of the VAD dimer with one monomer in orange and one in blue. (**b**) Surface representation clipped to highlight the tunnel at the monomer-monomer interface leading to the catalytic cavity. (**c-d**) Superposition between the substrate-binding sites in the wild-type enzyme and P151L without (**c**) and with (**d**) eugenol (green carbons). FAD carbons are in yellow, wild-type carbons in orange, P151L carbons in light blue. For the sake of clarity, the ribityl group of FAD is not shown.

**Figure 8:**
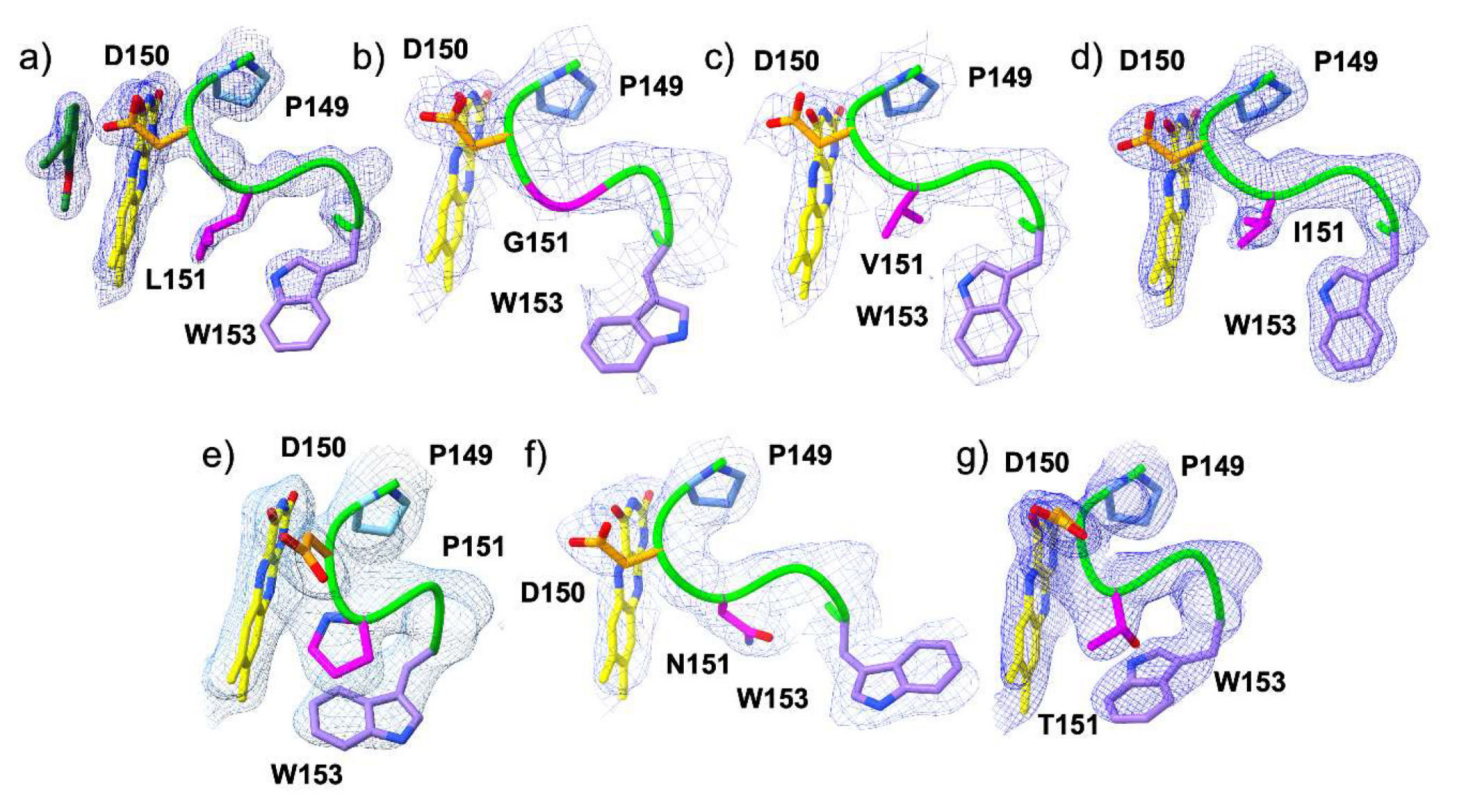
The conformation of the N5-interacting loop (residues 149-153) in VAD. The oxygen-reacting (**a**) P151L (1.80 Å resolution), (**b**) P151G (2.50 Å), (**c**) P151V (2.10 Å), (**d**) P151I (2.40 Å) enzymes are in the top raw. The poorly oxygen-reacting (**e**) wild type (2.35 Å), (**f**) P151N (2.10 Å), and (**g**) P151T (1.90 Å) VAD structures are at the bottom (Table 2). The carbons of Pro149, Asp150, residue 151, and Trp153 side chains are colored in light blue, orange, magenta and violet, respectively. The loop makes a sharp turn in the wild-type and P151T structures thanks to the *cis* conformation of residue 151. The weighted 2Fo-Fc maps are contoured at 1.2-1.4 σ levels.

To pinpoint structural differences induced by Pro151 mutations, we crystallized VAD and solved its structure (Table S1a-b). With 46% sequence identity and a root mean square deviation of 0.89 Å for the Cα atoms, its structure closely resembles that of VAO (Figure S2). Conserved features include the dimeric arrangement, a large chamber at the subunit interface, a constellation of active site Arg and Tyr residues, and the substrate binding mode in front of the flavin *si* side (Figure 7a-d). Pro151 is located within a sharp loop that covers the flavin edge on the *re* side of the cofactor ring. The residue is in the *cis* conformation with its carbonyl oxygen atom pointing towards the flavin N5 atom (Figure 7c). The crystal structures of the six P151 mutants were also solved (Table S1a-b). They are closely similar to the wild-type regarding the substrate-binding residues whereas loop 150-153 locally undergoes localized rearrangements, with each mutant adopting a distinct conformation. In general, the oxidase-active variants (P151L, P151G, P151I, P151V; Figure 8a-d) all adopt the *trans* conformation that slightly shifts the amino acid at position 151 away from the flavin. Conversely, in the dehydrogenase variants (P151N and P151T), like in the wild-type structure, the loop obstructs the access to the N5 atom (Figure 8e-g). In particular, Thr151 of P151T adopts a *cis* conformation as observed for the wild-type Pro151.

**Figure 9:**
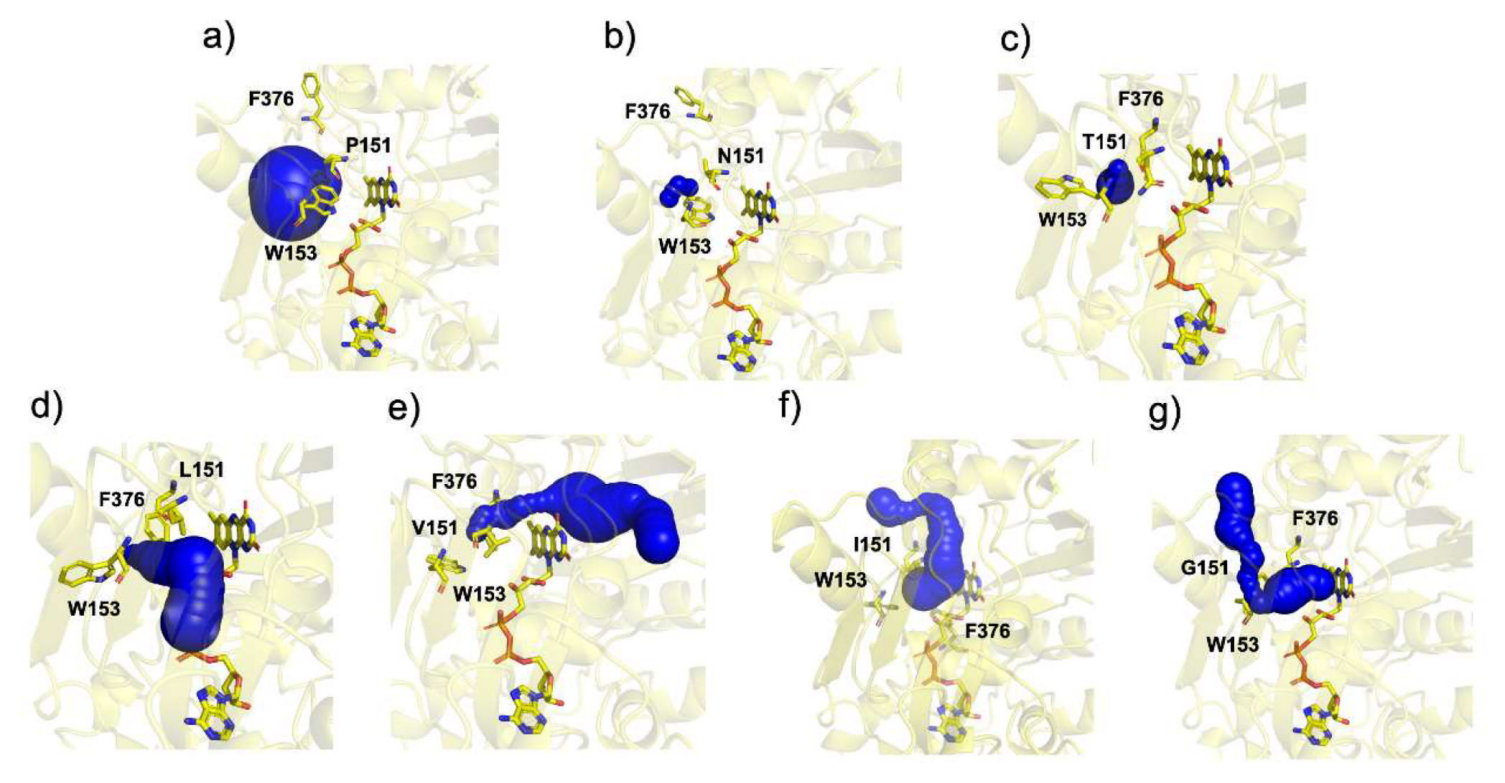
Accessibility of the flavin N5-C4a locus in VAD. The flavin ring is not accessible from the *re* side in the (**a**) wild type, (**b**) P151N, and (**c**) P151T VADs whereas a tunnel leading to the flavin *re* side is present in (**d**) P151L, **(e**) P151V, (**f**) P151I, (**g**) P151G. Calculated and displayed by the Caver plugin in PyMol.

To visualize the effects of this localized conformational changes on flavin accessibility, the VAD structures were analyzed with Caver, using the coordinates of residue 151 as the starting point (Figure 9) [25]. The analysis revealed a consistent pattern: in the oxidase mutants, the 150-153 loop conformations create tunnels that lead to the flavin *re* side. Conversely, no tunnels are present in the wild type and the dehydrogenase mutants where the flavin *re* face remains fully shielded by residue 151 and Trp153. These findings suggest that small, highly localized conformational changes can finely tune flavin accessibility. Specifically, subtle structural alterations can create or obstruct secondary pathways that might facilitate the diffusion of oxygen and water to the flavin N5 locus when a ligand occupies the substrate site on the flavin *si* side.

### Molecular dynamics highlight the substrate site as the main route for oxygen binding

**Figure 10:**
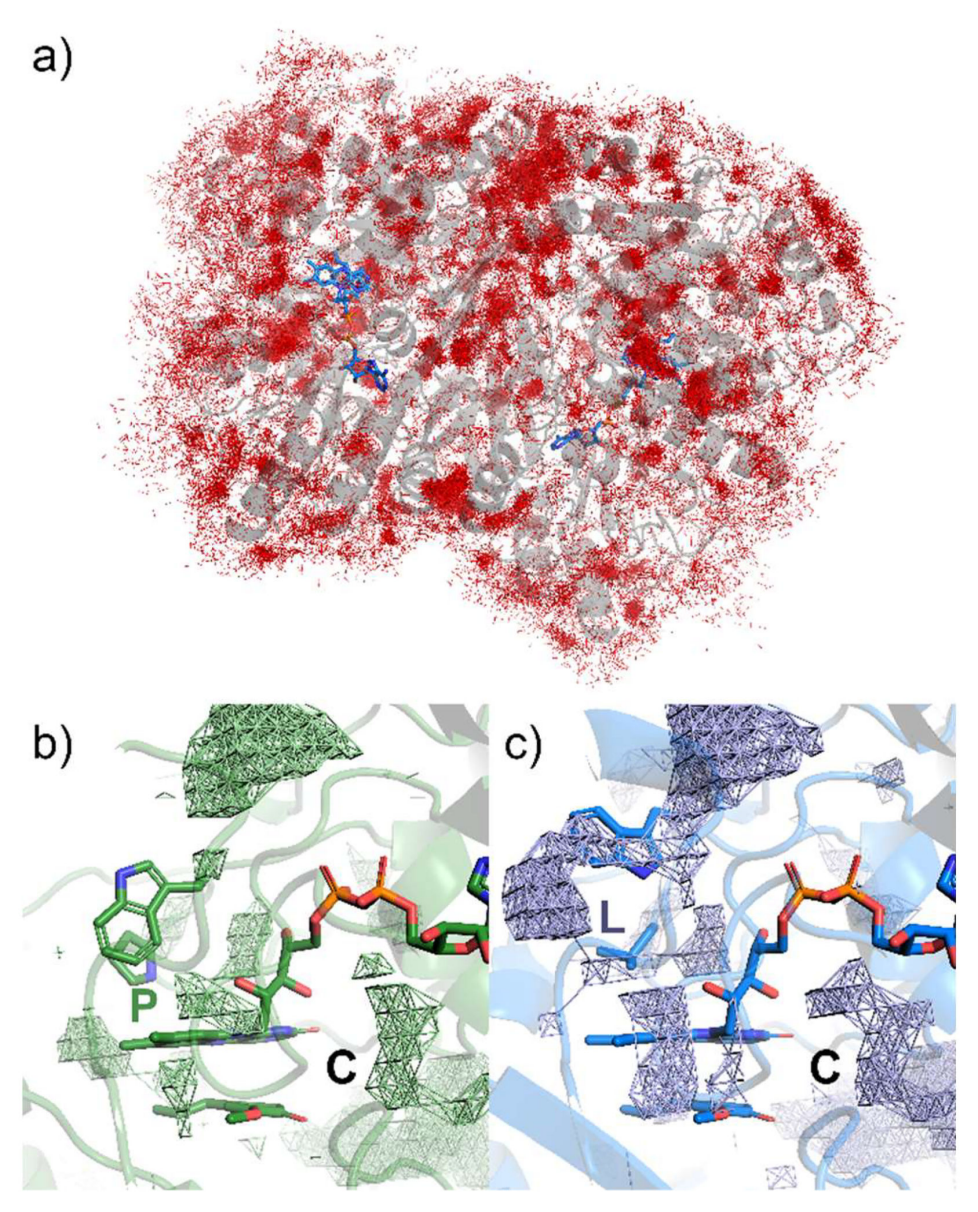
Oxygen diffusion and available cavities from MD simulations. (**a**) Cumulative, time-independent distribution of all O_2_ molecules arriving within <2.5 Å from any atom of P151L VAD (grey ribbon). The FAD in each protomer is represented as sticks in blue, while individual O_2_ molecules as red lines. (**b**,**c**) MDpocket analysis of (**b**) wild type and (**c**) P151L VAD. “P” and “L” represent the mutation site in the wild type and P151L, respectively, while “C” denotes the position closest to the large chamber at the monomer-monomer interface (Figure 7b).

The impact of the P151L mutation on oxygen diffusion and flavin accessibility was investigated through molecular dynamics simulations conducted at a 100-fold experimental O_2_ concentration and for 500 ns of simulation time, using modelled complexes of the reduced enzyme with the *p*-quinone methide intermediate [27]. The simulations revealed significant diffusion of O_2_ molecules, which approach well within 2.5 Å of the protein atoms in both the wild-type and P151L mutant structures (Figure 10a). These oxygen molecules are able to access and migrate through relatively hidden cavities, including the active site. MDpocket analysis of the molecular dynamics trajectories [34] confirmed that P151L possesses an elongated cavity that extends towards the flavin *re* side. This cavity is less extended in the wild-type enzyme due to the closer conformation of residues P151 and W153, as previously described (Figure 10b-c). Aside from these structural differences, no oxygen molecules were found to approach closer than 3.5 Å to the reactive flavin atoms N5-C4a in either structure. Therefore, the simulations support the notion that a ligand occupying the substrate site effectively shields the *si* side of the flavin from dioxygen. Meanwhile, the *re* side of the flavin does not serve as a highly accessible or efficient entry point for oxygen, even in the P151L mutant. The tunnel observed in the crystal structure of P151L may rather represent an auxiliary route for oxygen diffusion as proposed for VAO where a topologically similar tunnel has been described [41].

### Covalent flavinylation has no impact on oxygen reactivity

**Figure 11:**
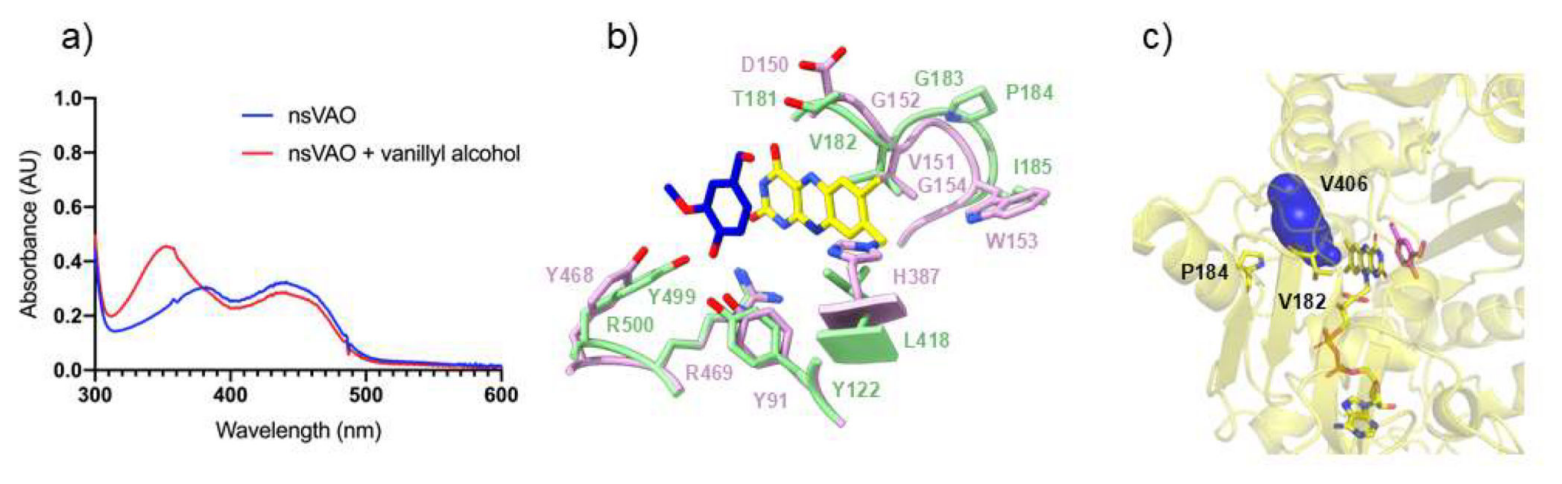
Properties of nsVAO. (**a**) nsVAO was incubated with vanillyl alcohol (45 mM). The spectra before substrate addition and after 30-minute incubation are in blue and red, respectively. Vanillin forms upon vanillyl alcohol oxidation (Fig. 2h). (**b**) Structural comparison between P151V VAD and wild type nsVAO (room mean square deviation of 0.94 Å for the Cα atoms). Vanillyl alcohol bound to wild type nsVAO is shown with blue carbons. Differently from VAD (Fig. 7b), the substrate in nsVAO positions its methoxy group above the flavin pyrimidine ring. Wild-type nsVAO carbons are in green, P151V VAD carbons in pink, FAD carbons are in yellow. For the sake of clarity, the ribityl group of FAD is not shown.

The enzyme from *Novosphingobium* sp. was of particular interest because it was predicted to lack a covalent flavin and to feature an unusual Val/Thr pair around the flavin N5 site, beside sharing 43% and 36% sequence identity with VAD and VAO, respectively (Table 1; Figure S2). The protein was expressed in high yields in *E. coli* and is thermally stable, with a melting temperature of 67 °C. Although it belongs to the EUGH clade, the enzyme is poorly active on eugenol whereas it converts vanillyl alcohol, with a *k*_*cat*_ of 0.29 s^-1^ (Figure 11a, Table 2). Moreover, its activity does not increase with the use of bovine heart cytochrome c as an artificial electron acceptor. The same properties were exhibited also by the T181D variant mutant, targeting the threonine residue that is typically an aspartate in the known sequence-related oxidases (Table 1-2; Figure 1b). The enzyme was therefore named as nsVAO, although we cannot exclude that it might use an endogenous cytochrome rather than oxygen as physiological substrate (Table S2). Its crystal structure confirms that FAD is non-covalently bound, supporting the notion that removal of the covalent flavin linkage does not drastically affect the properties of VAO/PCMH enzymes (Table 1, Figures S9-S10) [35,42]. The protein exhibits a narrow tunnel leading of the flavin *re* side where Val181, homologous to Pro151 of VAD, is in direct contact with the N5 atom (Figure 11b-c). The ability of nsVAO to use oxygen as electron acceptor thereby aligns with the oxidase activity observed for the P151V mutant of VAD (Table 2).

### The flavin microenvironment generally modulates oxygen reactivity in 4-phenol oxidizing enzymes

**Figure 12:**
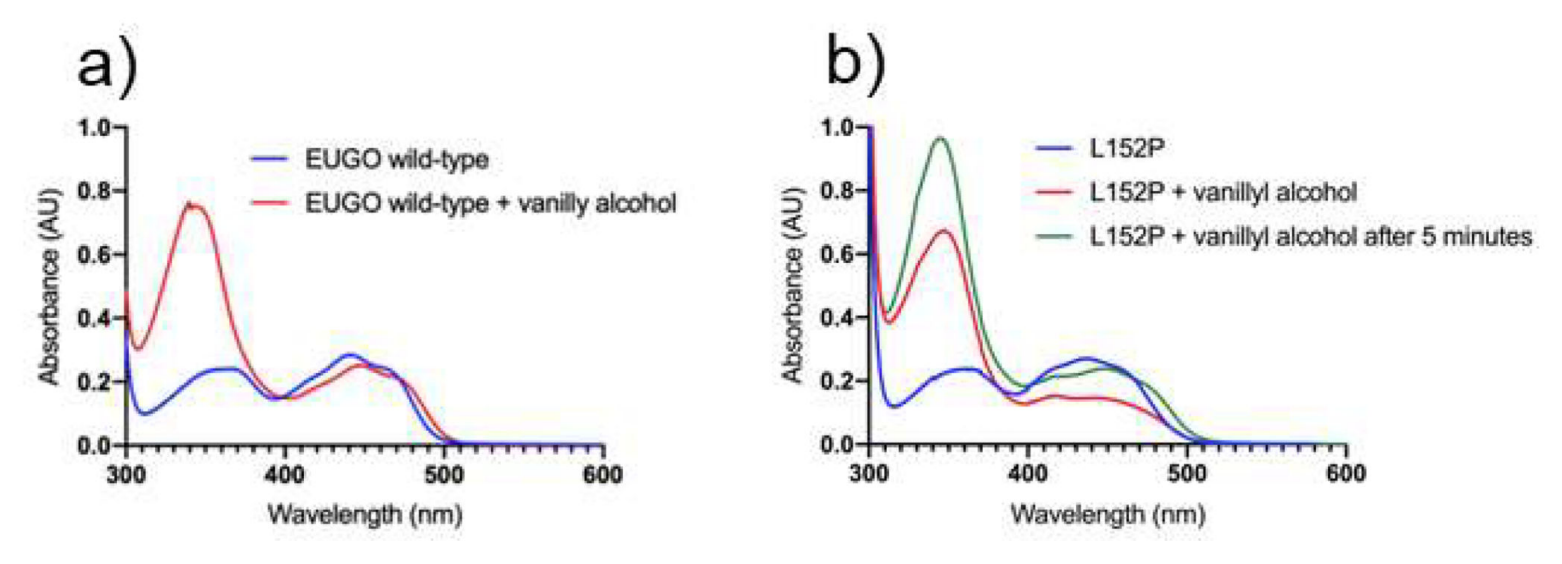
Spectral changes observed upon aerobic mixing of EUGO with vanillyl alcohol and dithionite. (**a**) No accumulation of reduced wild-type EUGO is observed when the enzyme is aerobically incubated with vanillyl alcohol. The spectra before substrate addition and after one-minute incubation are in blue and red, respectively. (**b**) EUGO L152P (blue) is reduced by the addition of the substrate (red, one-minute incubation) and complete re-oxidation is observed only after 5 minutes (green). Protein and vanillyl-alcohol concentrations are 30 μM and 45 μM, respectively. Vanillin forms upon vanillyl alcohol oxidation (Fig. 2h).

To finally verify that the N5-interacting position is a critical and general determinant of the oxygen reactivity, we studied the well-characterized oxidase EUGO from *Rhodococcus jostii* RHA1 [9]. Its N5-interacting residue was mutated by introducing a leucine-to-proline substitution (L152P, Table 1). Remarkably, the mutant enzyme displays lower *k_cat_* value when compared to the its respective wild-type form (0.02 s^-1^ *vs* 9.7 s^-1^; Table 2). Critically, its activity is partly restored by using DCPIP as electron acceptor (0.6 s^-1^). This indicates a shift in the catalytic behavior towards a dehydrogenase-type of function, a conclusion confirmed by the spectroscopic analysis (Figure 12). These results reinforce the concept that oxidase and dehydrogenase functionalities are interchangeable and primarily dictated by the flavin microenvironment.

## Discussion

Our work strongly indicates that the ability of VAO/PCMH enzymes to use molecular oxygen as acceptor depends on the accessibility to the flavin cofactor. FAD is deeply embedded within the protein and anchored by a covalent bond. The substrate binds within a cavity stabilized by a conserved set of residues, which enhance its reactivity by stabilizing its phenolate form (Figure 7c). The cavity is connected to a large chamber formed at the interface of the two enzyme subunits, creating a broad corridor for ligand diffusion and binding (Figure 7b). Substrate oxidation occurs through the transfer of a hydride from the Cα atom of the substrate side chain to the N5 atom of the flavin. This catalytic step requires precise positioning of the substrate, which stacks against the *si* face of the flavin, effectively shielding it (Figures 7d, 11a). The flavin *re* face is surrounded by protein residues; specifically, a loop (residues 150-153 in VAD) forms a sharp turn, positioning its tip at the reactive N5-C4a edge of the cofactor ring (Figure 8). The side chains of this loop are tightly packed against the flavin, obstructing access to the *re* side of the cofactor. As a result, the primary route for oxygen diffusion - an oxidant that reacts with the N5-C4a atoms of the flavin - is through the substrate cavity and a bound ligand may interfere with oxygen binding and reactivity. The reaction with oxygen can thereby be inhibited by the slow release of the quinone intermediate or the enzymatic product. Alternatively, the reduced enzyme may quickly release its product, allowing a new substrate molecule to bind before oxidation occurs. Our data indicates that this “substrate re-binding” mechanism contributes to the suppression of the oxygen reactivity in the dehydrogenases, where flavin oxidation indeed relies on electron transfer to an external cytochrome acceptor (Figures 3-4; Table 4). In contrast, in the oxidases, oxygen reactivity can be facilitated by the presence of alternative pathways for oxygen diffusion, decreased retention of the quinone methide intermediate, and/or less effective substrate re-binding to the reduced enzyme (Figures 9, 10 b-c, 11c-f). These processes are largely governed by the conformation and side-chain composition of the flavin microenvironment, which can modulate binding affinities, ligand exposure to water, the geometry of ligand binding, and access to the flavin via alternative routes that bypass the substrate cavity. The interplay between substrate binding and the local flavin environment explains how the function of VAO/PCMH enzymes can evolve through site-specific amino acid substitutions that define different enzyme clades. The local microenvironment around the flavin should therefore be the prime target for engineering functionally switched enzymes.

## Supplementary Material

The Supporting Information is available free of charge and contains: annotated phylogeny of the VAO/PCMH flavoprotein family; sequence alignment and identities; determination of the midpoint redox potential of VAD, steady-state and fast kinetics experiments on VAD, nsVAO and EUGO; crystallographic data. The raw data for the kinetics and spectroscopic experiments as well as the molecular dynamics input files, topology and coordinates of wild type VAD after minimization, topology and coordinates of P151L mutant VAD after minimization are available as separate files.

Supplementary Information

## Figures and Tables

**Table 1:** FAD-binding residues in representative 4-phenol oxidizing enzymes

Enzyme	Source	GenBank	Covalent FAD	N5- interacting	
VAO	*Penicillium simplicissimum*	P56216.1	H422	D170	L171
EPO	*Gulosibacter chungangensis*	WP_158051316.1	H395	E152	L153
EUGO	*Rhodococcus jostii* RHA1	Q0SBK1	H390	D151	L152
VAD	*Marinicaulis flavus*	WP_104830661.1	H387	D150	P151
EUGH	*Pseudomonas* sp.	Q8L385	Y381	T153	I154
nsVAO	*Novosphingobium* sp.	WP_124809348.1	L409/L418	T181	V182
PCMH	*Pseudomonas putida*	P09788.3	Y384	A154	P155

VAD is vanillyl-alcohol dehydrogenase, EUGH is eugenol hydroxylase, PCMH is *p*-cresolmethyl hydroxylase, VAO is vanillyl-alcohol oxidase, EUGO is eugenol oxidase, EPO is 4-ethylphenol oxidase (Figure 1; the sequences are aligned in Figure S2).

**Table 2:** Steady-state kinetics

	Substrate	*k*_cat_ (s^-1^)	K_M_^substrate^ (μM)	*k*_cat_/K_M_ (s^-1^ mM^-1^)	*k*_cat_ (s^-1^)	K_M_^substrate^ (μM)	*k*_cat_/K_M_ (s^-1^ mM^-1^)
**VAD** ^a^		**Molecular oxygen** ^b^			**2,6-Dichlorophenolindophenol** ^c^		
WT	Vanillyl alcohol	Inactive	-	-	0.44±0.02	74.2±12.4	6
WT	Eugenol	Inactive	-	-	0.81±0.06	51.2±13.1	16
WT	4-Methoxy methylphenol	Inactive	-	-	0.06±0.003	330±40	0.2
WT	4-Hydroxy benzylalcohol	Inactive	-	-	0.25±0.01	208±9	1.2
P151L^d^	Vanillyl alcohol	0.83±0.05	132±30	6	0.76±0.02	72.4±6.3	10.5
P151V	Vanillyl alcohol	0.09±0.01	174±20	0.52	0.90±0.04	42.2±6.6	21
P151I	Vanillyl alcohol	0.24±0.01	159±25	1.5	0.73±0.04	37.5±9.2	19
P151G	Vanillyl alcohol	0.86±0.02	60±9	14	1.49±0.07	316.5±39.7	5
P151N	Vanillyl alcohol	Inactive	-	-	0.14±0.07	151±24	1
P151T	Vanillyl alcohol	Inactive	-	-	0.48±0.03	17.6±5.5	27
**nsVAO** ^e^		**Molecular oxygen** ^b^			**Bovine heart cytochrome c** ^f^		
WT	Vanillyl alcohol	0.29±0.01	207±28	1.4	0.06±0.002	91.4±12.9	0.7
T181D	Vanillyl alcohol	0.28±0.01	203±22	1.4	0.07±0.004	337±67	0.2
**EUGO** ^g^		**Molecular oxygen** ^b^			**2,6-Dichlorophenolindophenol** ^c^		
WT	Vanillyl alcohol	9.68±0.43	204.4±28.9	47.4	0.48±0.017	17.6±3	27.2
P152L	Vanillyl alcohol	0.02±0.001	54±5.4	0.37	0.61±0.04	868±146	0.7

a Measured at 25 °C in 50 mM potassium phosphate pH 7.5 and 150 mM NaCl. Data are shown as average values ± standard deviation. n = 3 independent experiments. The kinetics plots are shown in Figure S4.

b Product formation was monitored spectroscopically.

c 2,6-Dichlorophenolindophenol (100 μM) reduction was monitored by following the absorbance at 600 nm. No activity by VAD was detected using bovine heart cytochrome c.

d Data for more substrates are reported in Table S3. No activity was detected using *p*-cresol.

e Measured at 35 °C in 50 mM Tris-HCl pH 9.0 and 300 mM NaCl. Data are shown as average values ± standard deviation. n = 3 independent experiments. The kinetics data are shown in Figure S5. nsVAO has low activities on eugenol and 4-hydroxybenzyl alcohol.

f Cytochrome c (50 μM) reduction was monitored by following the absorbance at 550 nm.

g Measured at 25 °C in 50 mM potassium phosphate pH 7.5 and 150 mM NaCl. Data are shown as average values ± standard deviation. n = 3 independent experiments. The *k*_*cat*_ and K_M_ values for wild-type EUGO are very similar to the previously reported values [9]. The kinetics plots are shown in Figure S6.

**Table 3:** The reductive half-reaction in VAD

	Substrate	*k*_red_ (s^-1^)	*K*_d_ (μM)
Wild type	vanillyl alcohol	0.50 ± 0.01	24.2 ± 3.3
P151L	vanillyl alcohol	339 ± 11	1060 ± 80

Reactions were performed anaerobically at 25 °C in 50 mM potassium phosphate pH 7.5, 150 mM NaCl. Data are shown as average values ± standard deviation. n = 3 independent experiments.

**Table 4:** The oxidative half-reaction in VAD.

	Reductant	1 mM vanillin	k_ox_ (M^-1^ s^-1^)
WT	vanillyl alcohol	-	26.9
	dithionite	-	1.4 x 10^3^
	dithionite	+	1.4 x 10^3^
P151L	vanillyl alcohol	-	1.5 x 10^3^
	dithionite	-	1.9 x 10^4^
	vanillyl alcohol	+	1.5 x 10^3^

Reactions were performed at 25 °C in 50 mM potassium phosphate pH 7.5, 150 mM NaCl

## Data Availability

Crystal structures of VADs and nsVAOs have been deposited under PDB, accession codes: 8S7P, 8S7U, 8S7Q, 8S7W, 8S7N, 8S7R, 8S7T, 8S7S, 9FFK, 9FGE.
