# Supplementary material for: Dehydrogenase versus oxidase function: the interplay between substrate binding and flavin microenvironment
Source: ACS Catal. Author manuscript; Available in PMC 2025 Jan 7. (PMC7617285; doi:10.1021/acscatal.4c05944)
Supplement: Supplementary Information [file EMS201890-supplement-Supplementary_Information.pdf]

Supporting Information

Dehydrogenase *versus* oxidase function: the interplay between substrate binding and flavin microenvironment

Teresa Benedetta Guerriere[1], Alessandro Vancheri[2], Ilaria Ricotti[2], Stefano A Serapian[2], Daniel Eggerichs[3], Dirk Tischler[3], Giorgio Colombo[2], Maria L. Mascotti[4], Marco W. Fraaije[5*], Andrea Mattevi[1*]

[1]Department of Biology and Biotechnology "Lazzaro Spallanzani", University of Pavia, Pavia, Italy 27100.

[2] Department of Chemistry, University of Pavia, 27100 Pavia, Italy

[3] Microbial Biotechnology, Ruhr University Bochum, 44780, Bochum, Germany.

[4] IHEM CONICET, Universidad Nacional de Cuyo, Mendoza, Argentina, M5502JMA.

[5] Molecular Enzymology Group, University of Groningen, Groningen, The Netherlands, 9747AG.

***Correspondence**: M.W.Fraaije@rug.nl; andrea.mattevi@unipv.it

Table of Contents

Annotated phylogeny of the VAO/PCMH flavoprotein family (Figure S1) S2
Sequence alignment and identities (Figure S2) S4
Determination of the midpoint redox potential (Em) of VAD (Figure S3) S6
Steady-state kinetics experiments on VAD (Figure S4) S7
Steady-state kinetics experiments on nsVAO (Figure S5) S9
Steady-state kinetics experiments on EUGO (Figure S6) S9
Stopped-flow kinetics (Figure S7) S10
Enzyme-monitored turnover experiment (Figure S8) S11
The electron density of the flavin and surronding residues in nsVAO (Figure S9) S12
Properties of nsVAO T181D mutant (Figure S10) S12
Data collection and refinement statistics (Table S1) S13
Genetic context (Table S2) S16
Steady state kinetic parameters of P151L VAD (Table S3) S18
Primers used for quick change mutagenesis (Table S4) S18

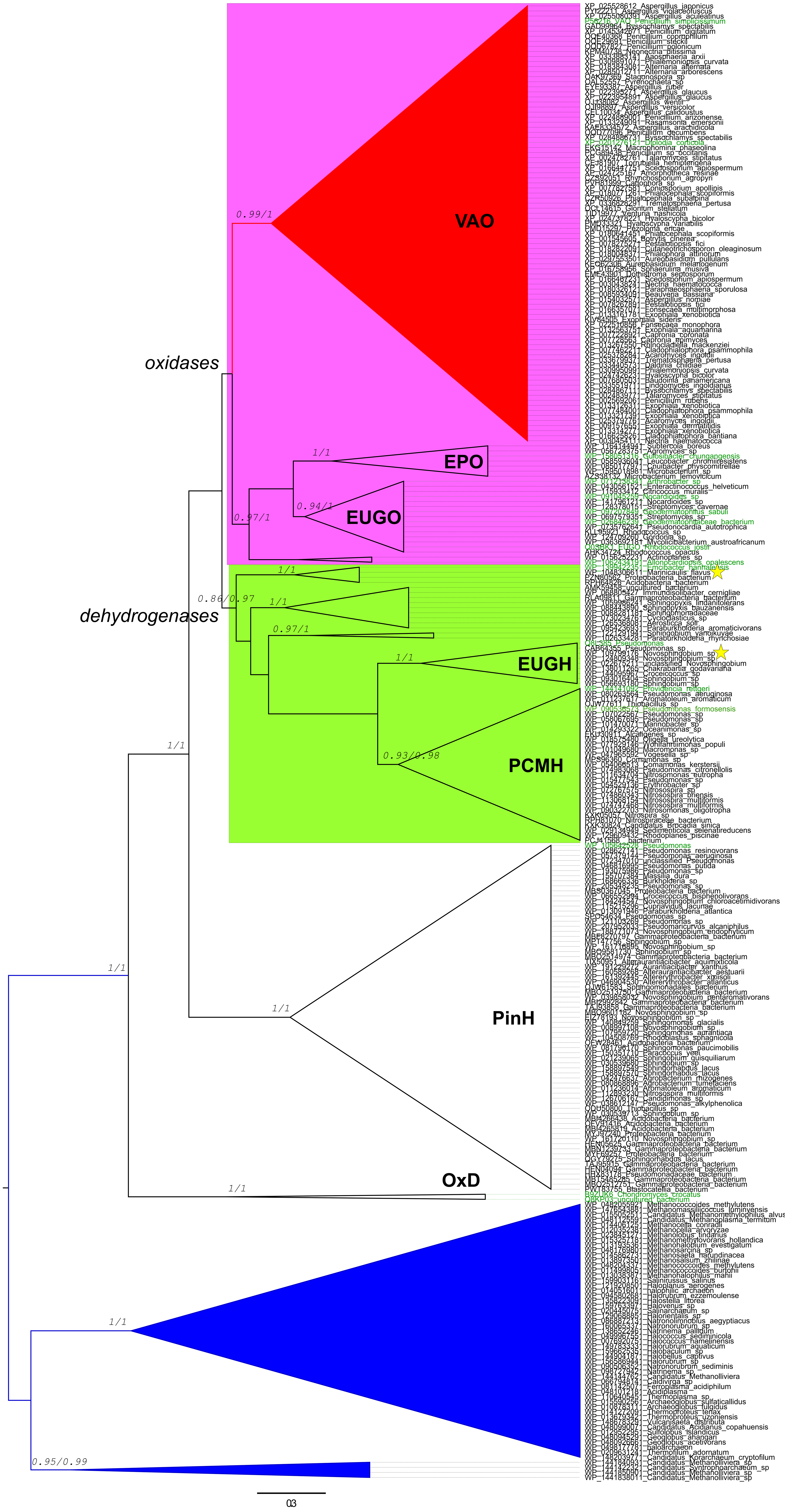

VAO
EPO
EUGO
EUGH
PCMH
PinH
OxD
oxidases
dehydrogenases
0.99/1
1/1
0.94/1
0.97/1
0.86/0.97
1/1
1/1
0.97/1
1/1
0.93/0.98
1/1
1/1
1/1
1/1
1/1
0.95/0.99
03

Supplementary Figure 1. Annotated phylogeny of the VAO/PCMH flavoprotein family. The tree was constructed in RAxML v8.2.10 using 500 rapid bootstraps and automated model selection. The Transfer Bootstrap Expectation/Poster Probability support values are shown at the nodes. Taxonomic groups are colored as follows: Fungi (red), Bacteria (white) and Archaea (blue). The accession codes and species name are given for each sequence included in the dataset. Experimentally characterized enzymes are shown in green. Clades are named as in the main text. The two sequences characterized in this work are marked with yellow stars. The scale bar indicates substitutions per site.

```
PinH        ----------------------------------MLPPGIDSATFQKALSAFANVVGKEW     26
PCMH        ---------------------------MSEQNNAVLPKGVTQGEFNKAVQKFRALLGDDN     33
EUGH        -----------------------------MDTTVMLPAGVSTEQFSKAIAEFRLLLGEDN     31
nsVAO       MSGFSIDRRSLLGTGLGASLAAGLGTGASAANPAPLAPGRTKADFAGAMKAFRGVVGAEW     60
VAO         ------------------------MSKTQEFRPLTLPPKLSLSDFNEFIQDIIRIVGSEN     36
VAD         -------------------------------MAAPLPEGVSAEAMSSALDRFARIVGADW     29
EPO         ------------------------------MNFRTLPDGVSAEQFANAISEFSETIGSEY     30
EUGO        -------------------------------MTRTLPPGVSDERFDAALQRFRDVVGDKW     29
                                               *        :   :  :   :* .

PinH        VFTSDEDLKL----------YRDAYSPYMGEAEERLASAAVAPDTSEQVQEIARIANQYS     76
PCMH        VLVESDQLVP----------YNKIMMPVEN--AAHAPSAAVTATTVEQVQGVVKICNEHK     81
EUGH        VLATAERVVP----------YTKLLIPTED-DASFMPSGALTPGSVEEVQKILAICNRYK     80
nsVAO       VFGDEEAVVP----------WSKTYIPDP--AHQYKPVGAVCPQSVEEVQEIVRIANTYK     108
VAO         VEVISSKDQIVDGSYMKPTHTHDPHHVMD--QDYFLASAIVAPRNVADVQSIVGLANKFS     94
VAD         VFT-EDKITP----------YEDPYTISND-ETEHRPYAAVAPASTEEVQEIVRVANEFG     77
EPO         VRVDEATVSE----------YDDKFPVTDG--DEFKGSAVIWPGSTEDVQVIVRIANKYG     78
EUGO        VLSTADELEA----------FRDPYPVGAA--EANLPSAVVSPESTEQVQDIVRIANEYG     77
            *                     .               . :   .  :** :  :.* .

PinH        IPLYTISTGKNLGYGGSAPTYSGSVVLDL-KRMNRIIEVNEKQAYCIVEPGVSYFDMYRY     135
PCMH        IPIWTISTGRNFGYGSAAPVQRGQVILDL-KKMNKIIKIDPEMCYALVEPGVTFGQMYDY     140
EUGH        LPVWPISTGRNWGYGSASPATPGQLILDL-RKMNKIIEIDVDSCTALLEPGVTYQQLYDH     139
nsVAO       QPLWTVSTGKNMGYGMTAPATPGQVVLDL-KRMNRILEVDADLGTCLLEPGVTYQQLKDY     167
VAO         FPLWPISIGRNSGYGGAAPRVSGSVVLDMGKNMNRVLEVNVEGAYCVVEPGVTYHDLHNY     154
VAD         VPLWPVSRGKNFAYGGAAPVMSGTVVLDM-NRMNRILEVNEEFGYALVEPGVSYFELYDY     136
EPO         IPLHAFSGGRNLGYGGSSPMLTGTVLLHLGKRMNRVLEINEKLAYAVVEPGVDYKTLYEA     138
EUGO        IPLSPVSTGKNNGYGGAAPRLSGSVIVKTGERMNRILEVNEKYGYALLEPGVTYFDLYEY     137
             *:  .* *:* .** ::*   * :::.  ..**::::: .   .::**** :  :

PinH        LQEKK--SKLWLDVPDPGW-GSMVGNAMDRGAGYTAAQFRNHFDAHCGMEVVLANGEVMR     192
PCMH        IQENN--LPVMLSFSAPSAIAGPVGNTMDRGVGYTP--YGEHFMMQCGMEVVLANGDVYR     196
EUGH        LKENN--IPLMLDVPTIGPMVGPVGNTLDRGVGYTP--YGEHFMMQCGMEVVLANGEILR     195
nsVAO       LEEHK--IPLWIDVPTVGPIASPVGNTLDRGVGYTP--YGEHFMFQCGMEVCLPDGRLMR     223
VAO         LEANNLRDKLWLDVPDLGG-GSVLGNAVERGVGYTP--YGDHWMMHSGMEVVLANGELLR     211
VAD         IQEKG--LKLWIDVPDPGW-GSVVGNALDHGIGYTP--YGDHFAMQCGMEVVLPNGEVVR     191
EPO         VRDSG--AKLMIDPAELDW-GSVMGNTMEHGVGYTP--YADHSMWRCGMEVVLADGEVLR     193
EUGO        LQSHD--SGLMLDCPDLGW-GSVVGNTLDRGVGYTP--YGDHFMWQTGLEVVLPQGEVMR     192
            :.       : :.    .   . :**::::* ***   : :*   : *:** * :* : *

PinH        TGMGAMPKS----------------KTWAMYKTGFGPAIDGIFSQSNFGIVTKMGFWLMP     236
PCMH        TGMGGVPGS----------------NTWQIFKWGYGPTLDGMFTQANYGICTKMGFWLMP     240
EUGH        TGMGSVPKA----------------KTWQAFKWGYGPYVDGIFTQSNFGVVTKMGLWLMP     239
nsVAO       TGMGSIKGS----------------TAWQAFKWGYGPYLDGLFTQSNFGIVTKMGLWLMP     267
VAO         TGMGALPDPKRPETMGLKPEDQPWSKIAHLFPYGFGPYIDGLFSQSNMGIVTKIGIWLMP     271
VAD         TGMGAMPGN----------------NTWQLFKYGYGPYVDGIFSQSNFGVVTKMGIWLMP     235
EPO         TGMGGLPGS----------------EAWHLYPGQLGPSIEGLFEQSNFGICTRMGMQLMP     237
EUGO        TGMGALPGS----------------DAWQLFPYGFGPFPDGMFTQSNLGIVTKMGIALMQ     236
            ****.:                        :    **  :*:* *:* *:  *::*: **

PinH        EPEAFLKGHIHLSQYSDMVPLVELMTELENSK-IFTGYPDINSPAMGTPS-LAGLHEFLA     294
PCMH        KPPVFKPFEVIFEDEADIVEIVDALRPLRMSN-TIPNSVVIASTLWEAGSAHLTRAQYTT     299
EUGH        KPPVIKPFMVRYQNESDVVKAIDAMRPLRINQ-LIPNVVLFMHGHYETAI-CKTRAEVTS     297
nsVAO       KPPVYKPFMVRHANMEDVPKIIEAMRPLRVSN-LVANCNLMMSASYQLAM-FKRRNEIVP     325
VAO         NPRGYQSYLITLPKDGDLKQAVDIIRPLRLGM-ALQNVPTIRHILLDAAV-LGDKRSYSS     329
VAD         EPAGYRPYLITFENEDDIETVTERLRPLKVA-GVIQNGATVRSLVLDAAI-TRTKSQYYD     293
EPO         TPPEMLSFAIYFENEDDLPAIMETTLPLRIGMAPLQAAPIVRNVTFDAAC-VSKREEWQT     296
EUGO        RPPASQSFLITFDKEEDLEQIVDIMLPLRINMAPLQNVPVLRNIFMDAAA-VSKRTEWFD     295
             *       :   .  *:    :    *.     .     .               .
```

```
PinH            HGPKEQDPEFMALLGRGAKPEEYEAYAKKKGIPFWTCALTFYGPEKVIRAQWEYAQERFK    354
PCMH            -E------------PGHTPDSVIKQMQKDTGMGAWNLYAALYGTQEQVDVNWKIVTDVFK    346
EUGH            -D------------PGPISEPDARKVFKELGIGYWNVYFALYGTAEQIAVNEKIVRSIIE    344
nsVAO           -D------------GAPLDEASLKKAASANGLGMWNTYFALYGTEQTVAGVEPIIRATLT    372
VAO             -R------------TEPLSDEELDKIAKQLNLGRWNFYGALYGPEPIRRVLWETIKDAFS    376
VAD             -G------------DGPIPPSVAKTMMADLDLGMWNFCGALYGPPPVMDTLWTAIRDSFA    340
EPO             -E------------PGPLTDEAKQRMVDELGIGHWIVYGTCYGPRWQIDKYIEMIRDAYL    343
EUGO            -G------------DGPMPAEAIERMKKDLDLGFWNFYGTLYGPPPLIEMYYGMIKEAFG    342
                                              .:  *    : **

PinH            KAFPDAKFAEHEFYKLPLTPEQA--EKVEYPAQFGIPNLRTFAIGARSNWNPAPPTHGHA    412
PCMH            KL-GKGRIVTQEEAG------DTQPFKYRAQLMSGVPNLQEFGL---YNWRG--G-GGSM    393
EUGH            PS--GGEIVTEAEAG------DNILFHHHKQLMCGEMTMEEMNI---YRWRG--AGGGAC    391
nsVAO           AS--GGEVLTAAEME------GNPWFHHHQTLMQGGLNLDEVGL---LRWRG--AGGGLA    419
VAO             AI-PGVKFYFPEDTP------ENSVLRVRDKTMQGIPTYDELKW---IDWLP--N-GAHL    423
VAD             DI-PGVKFYFPEDRR-----HKVDLLLHRAETMKGVPKLTEFNF---LNWDG--G-GGHV    388
EPO             QI-PGARFETNETLPLREGDRASELLNARHELNTGVPNRHSAAV---FDWFP--N-AGHF    396
EUGO            KI-PGARFFTHEER----DDRGGHVLQDRHKINNGIPSLDELQL---LDWVP--N-GGHI    391
                      ..                    .     *  .           *       .

PinH            WFSPVIPRDGAEVLKINEVLGTEARRLGIPLIFAMIVPVPSWERSFTFIIPLFISEDPAQ    472
PCMH            WFAPVSEARGSECKKQAAMAKRVLHKYGLDYVAEFIV---APRDMHHVIDVLYDRTNPEE    450
EUGH            WFAPVAQVKGQEAELQVKLAQRVLAKHNLDYTAGFAI---GWRDLHHIIDVLYDRNNAEE    448
nsVAO           WFAPVAAARGVEAERQTALAKEIVEKHGFDYTAAYAI---GWRDLHHIIALLFDKADPTQ    476
VAO             FFSPIAKVSGEDAMMQYAVTKKRCQEAGLDFIGTFTV---GMREMHHIVCIVFNKKDLIQ    480
VAD             GFSPVSPITGKDAIKQYNMVSSRVREYGFDYMGLLAI---GWRDLHHVTVIVYDKTDPDE    445
EPO             FYAPVSAPSGEDAAKQYEDTKRISDDHGIDYLAQFII---GLREMHHICLPLYDTADPAS    453
EUGO            GFSPVSAPDGREAMKQFEMVRNRANEYNKDYAAQFII---GLREMHHVCLFIYDTAIPEA    448
                 ::*:    * :               .        :   . .  . .   ::

PinH            NKRSREVFRHLIKVAADNGWGEYRTAPTFQADVMDTYSFGDHALLRFHESIKDAVDPKGI    532
PCMH            TKRADACFNELLDEFEKEGYAVYRVNTRFQDRVAQSYGPV---KRKLEHAIKRAVDPNNI    507
EUGH            KKRAYACFDELIEVFAAEGFASYRTNIAFMDKVAKKFGST---NMHVNQQIKKALDPNGI    505
nsVAO           EQKADACYRELVTRFGAQGWASYRTGVNSMDLVAQQYGEV---NRDFNRTLKRAIDPNGI    533
VAO             KRKVQWLMRTLIDDCAANGWGEYRTHLAFMDQIMETYNWNNSSFLRFNEVLKNAVDPNGI    540
VAD             RKKLDELFNILVDEAAAEGYGEYRTHIRYMDRIAKTYSWNDNALWKMHETIKDALDPNGI    505
EPO             RKETLDMTRELIRAGAEEGYGIYRAHNVLADQVAETYSFNNHIQRRSHERIKDALDPNGI    513
EUGO            REEILQMTKVLVREAAEAGYGEYRTHNALMDDVMATFNWGDGALLKFHEKIKDALDPNGI    508
                 ..       *:      *:. **.       :   :.         .. :* *:**:.*

PinH            LSPGRYGIWPKHLRDKHRYKGGDSV 557
PCMH            LAPGRSGIDLNNDF----------- 521
EUGH            LAPGKSGIHLPD------------- 517
nsVAO           LSPGKSGIHP--------------- 543
VAO             IAPGKSGVWPSQYSHVTWKL----- 560
VAD             LAPGKSGIWGKNRRKA--------- 521
EPO             LNPGKSGIWPERLRNK--------- 529
EUGO            IAPGKSGIWSQRFRGQNL------- 526
                : **: *:
```

	PinH	PCMH	EUGH	nsVAO	VAO	VAD	EPO	EUGO
PinH	100.00							
PCMH	34.71	100.00						
EUGH	32.02	53.79	100.00					
nsVAO	36.17	44.94	54.86	100.00				
VAO	37.96	32.43	35.21	36.36	100.00			
VAD	41.36	43.47	40.90	42.91	46.33	100.00		
EPO	36.85	35.47	36.45	36.27	41.84	41.92	100.00	
EUGO	41.43	39.49	38.94	39.96	46.45	51.45	51.53	100.00

Figure S2. Sequence alignment and identities of pinoresinol hydroxylase from *unclassified Pseudomonas* (PinH), *p*-cresolmethylhydroxylase from *Pseudomonas putida* (PCMH), eugenol hydroyxylase from *Pseudomonas* sp, (EUGH), vanillyl alcohol oxidase from *Novosphingobium* sp (nsVAO), vanillyl-alcohol oxidase from *Penicillium simplicissimum* (VAO), vanillyl-alcohol dehydrogenase from *Marinicaulis flavus* (VAD), 4-ethylphenol oxidase from *Gulosibacter chungangensis* (EPO) and eugenol oxidase from *Rhodococcus jostii* (EUGO). The critical residues, listed in **Table 1**, are highlighted

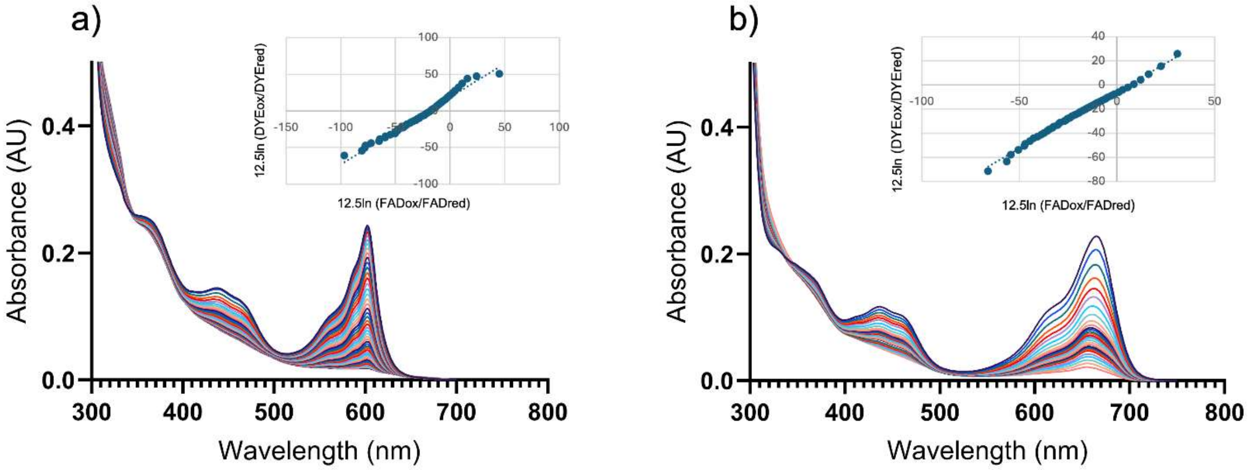

Figure S3. **Determination of the midpoint redox potential (Em) of VAD using the xanthine/xanthine oxidase method.** Spectral changes observed for the determination of the midpoint redox potential of (**a**) wild type VAD using methylene blue as dye; (**b**) P151L using thionine acetate as dye. The spectra show the simultaneous reduction of the protein and the dye. They were measured every minute for 60 minutes. The inset shows a plot in which the $\log[(Ox_{dye})/(Red_{dye})]$ is on the Y-axis, and $\log[(Ox_{enzyme})/(Red_{enzyme})]$ is on the X-axis. The redox potential of the wild-type enzyme and P151L was found to be +82±2 mV and +6±1 mV, respectively.

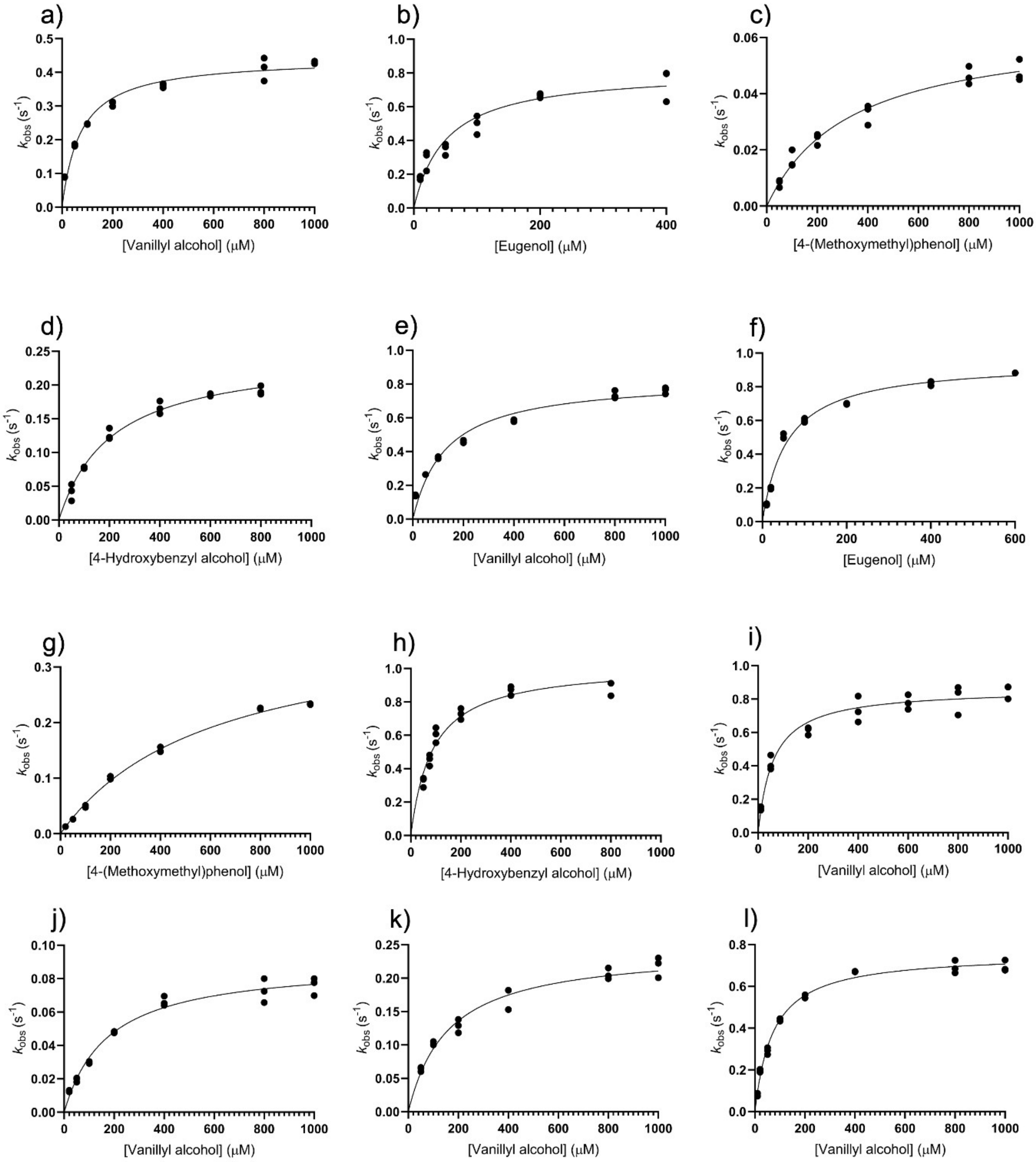
a)
k_{obs} (s^{-1})
[Vanillyl alcohol] (μM)
b)
k_{obs} (s^{-1})
[Eugenol] (μM)
c)
k_{obs} (s^{-1})
[4-(Methoxymethyl)phenol] (μM)
d)
k_{obs} (s^{-1})
[4-Hydroxybenzyl alcohol] (μM)
e)
k_{obs} (s^{-1})
[Vanillyl alcohol] (μM)
f)
k_{obs} (s^{-1})
[Eugenol] (μM)
g)
k_{obs} (s^{-1})
[4-(Methoxymethyl)phenol] (μM)
h)
k_{obs} (s^{-1})
[4-Hydroxybenzyl alcohol] (μM)
i)
k_{obs} (s^{-1})
[Vanillyl alcohol] (μM)
j)
k_{obs} (s^{-1})
[Vanillyl alcohol] (μM)
k)
k_{obs} (s^{-1})
[Vanillyl alcohol] (μM)
l)
k_{obs} (s^{-1})
[Vanillyl alcohol] (μM)

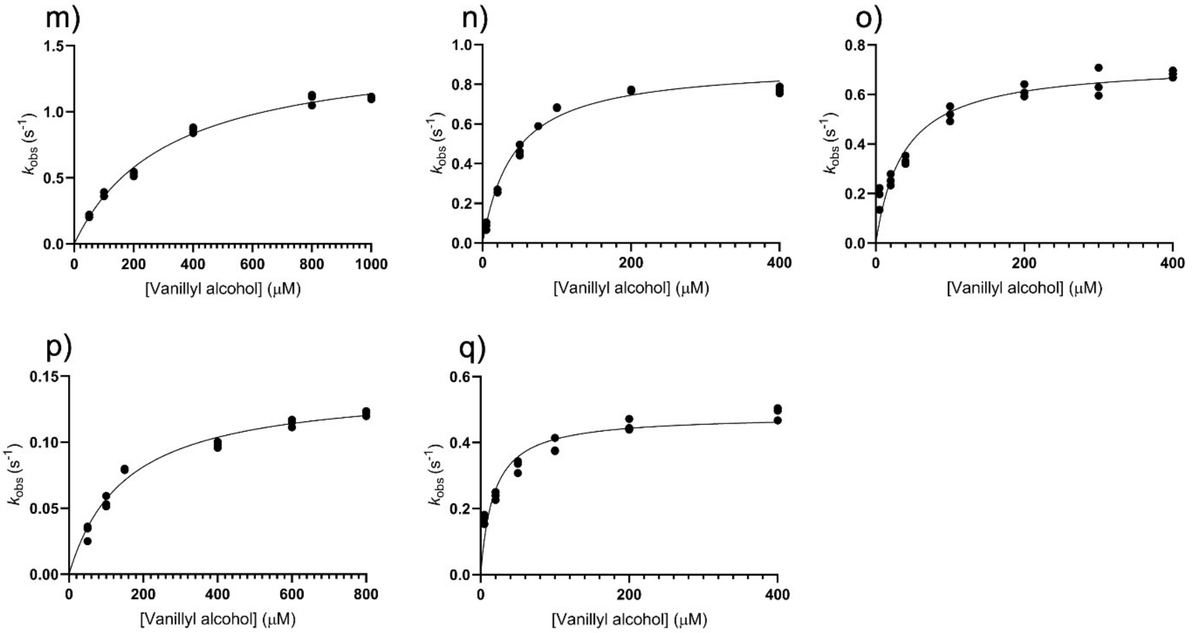

Figure S4. Steady-state kinetics experiments on VAD. Wild-type VAD with (**a**) vanillyl alcohol and 2,6-dichlorophenolindophenol (DCPIP), (**b**) eugenol and DCPIP, (**c**) 4-(methoxymethyl)phenol and DCPIP, (**d**) 4-hydroxybenzyl alcohol and DCPIP. P151L with (**e**) vanillyl alcohol, (**f**) eugenol, (**g**) 4-(methoxymethyl)phenol, (**h**) 4-hydroxybenzyl alcohol. (**i**) P151G with vanillyl alcohol. **j**) P151V with vanillyl alcohol. (**k**) P151I with vanillyl alcohol. (**l**) P151L with vanillyl alcohol and DCPIP. (**m**) P151G with vanillyl alcohol and DCPIP. (**n**) P151V with vanillyl alcohol and DCPIP. (**o**) P151I with vanillyl alcohol and DCPIP. (**p**) P151N with vanillyl alcohol and DCPIP. (**q**) P151T with vanillyl alcohol and DCPIP. n = 3 independent experiments, individually plotted as dots.

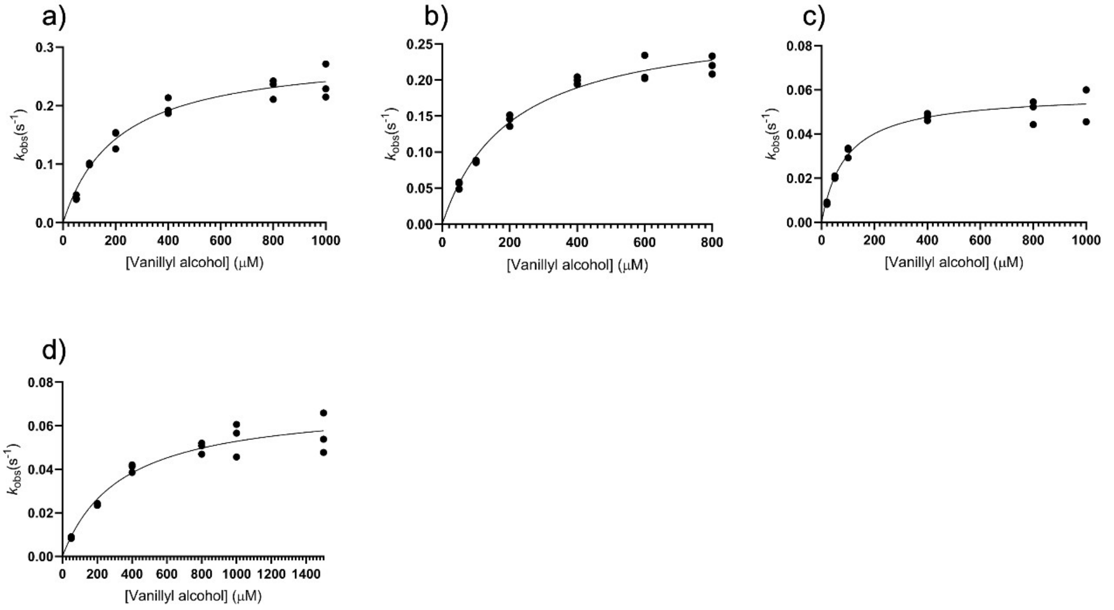

Figure S5. Steady-state kinetics experiments on nsVAO. (**a**) Wild type enzyme with vanillyl alcohol, (**b**) T181D with vanillyl alcohol, (**c**) wild type with vanillyl alcohol and cytochrome c. (**d**) T181D with vanillyl alcohol and cytochrome c. n = 3 independent experiments, individually plotted as dots.

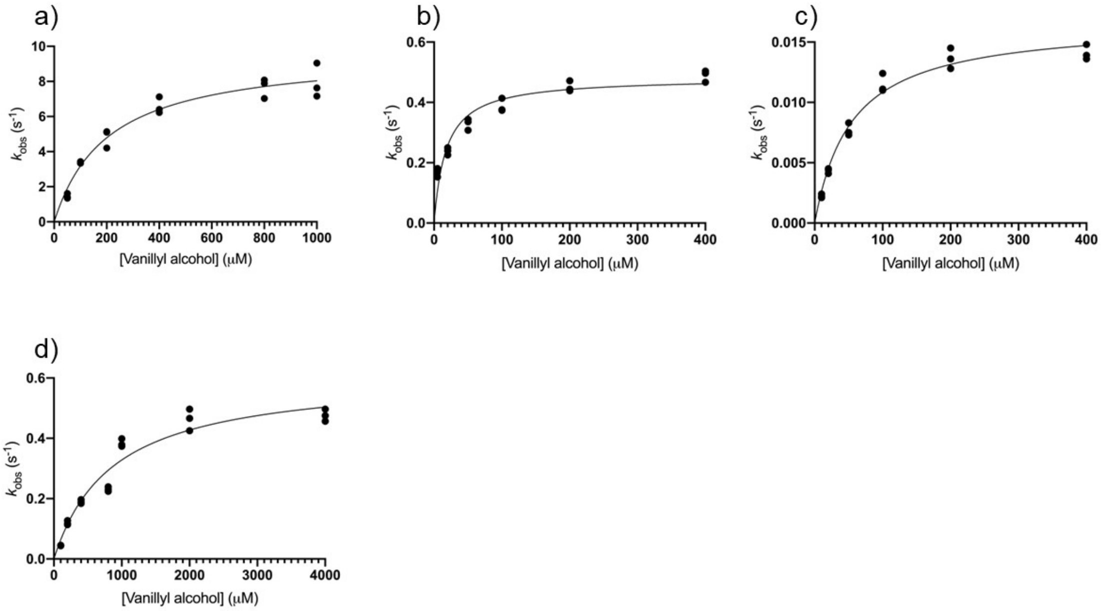

Figure S6. Steady-state kinetics experiments on EUGO from *Rhodococcus jostii* RHA1. (**a**) Wild type enzyme with vanillyl alcohol, (**b**) wild type with vanillyl alcohol and DCPIP, (**c**) EUGO L152P with vanillyl alcohol, (**d**) EUGO L152P with vanillyl alcohol and DCPIP.

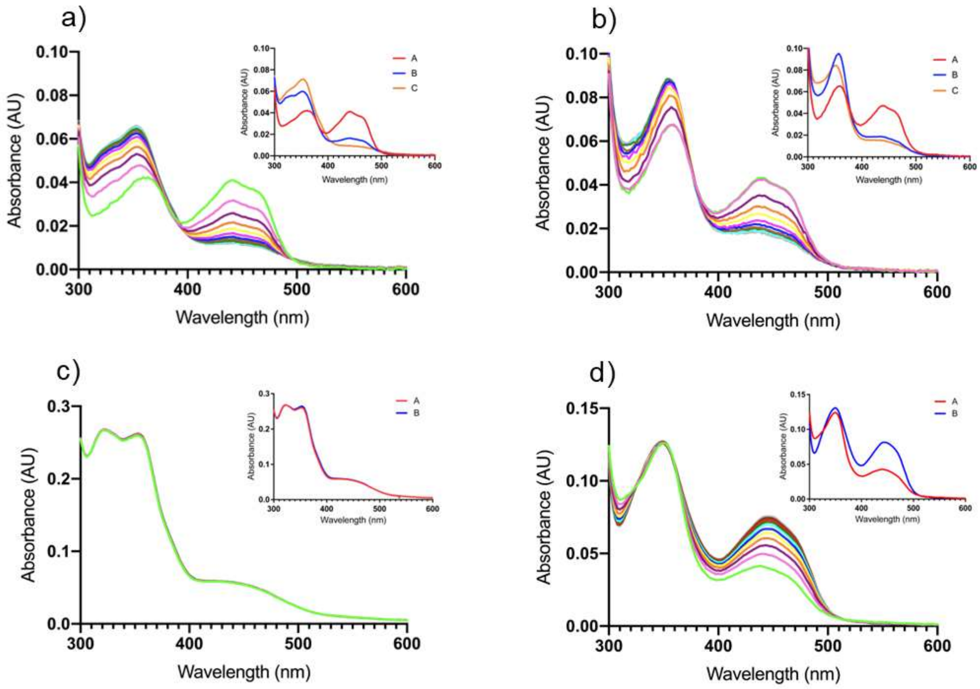

Figure S7. Spectral changes observed through stopped-flow apparatus. (**a**) Anaerobic mixing of wild-type VAD (5 µM) with vanillyl alcohol (400 µM) and **(b)** P151L (5 µM) with vanillyl alcohol (1000 µM). Spectral scans are shown from 0 to 10 s with intervals of 1 s for the wild-type enzyme, and from 0 to 20 ms with intervals of 2 ms for P151L. The data were fit with two exponential functions (*A→B, B→C)*. The *insets* show the deconvolution of the spectral scans for *A, B* and *C*. In the *B* state, the enzyme is almost completely reduced and the k_{red} values listed in **Table 3** refer to the *A→B* conversion rates. (**c**) Vanillyl alcohol-reduced wild-type VAD (20 µM) upon mixing with oxygen and (**d**) vanillyl alcohol-reduced P151L (10 µM) upon mixing with oxygen. Spectral scans are shown from 0 to 10 s with intervals of 1 s for the wild-type enzyme, and from 0 to 2 s with intervals of 0,2 s for P151L. The data were fit with one exponential function (*A→B)* as listed in **Table 4**. The *insets* show the deconvolution of the spectral scans for *A* and *B*.

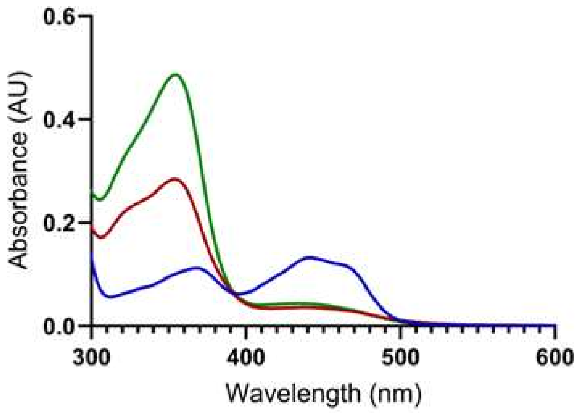

Figure S8. Enzyme-monitored turnover experiment. Spectral changes observed upon aerobic mixing wild type and P151L VAD (12 µM) with 2.5 mM vanillyl alcohol. The spectrum of the initial oxidized wild-type enzyme is in blue. The spectrum of P151L would be indistinguishable and is not shown for clarity. The spectra of the wild-type and P151L proteins after 10 s incubation are in red and blue, respectively.

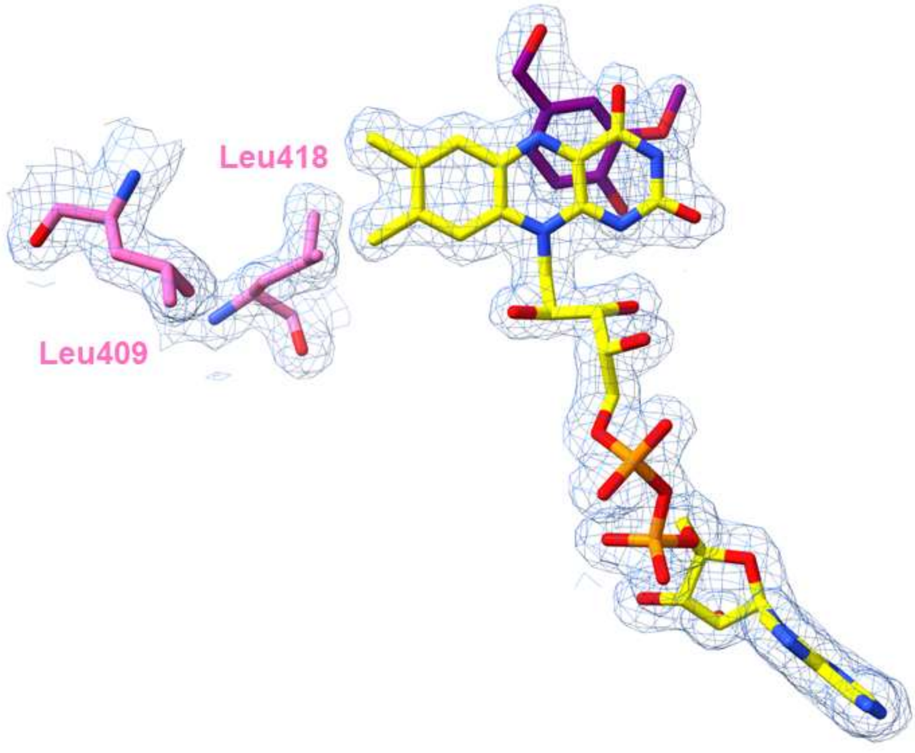

Figure S9. The electron density of the flavin and surronding residues in nsVAO. The weighted 2Fo-Fc map is contoured at 1.2 σ level.

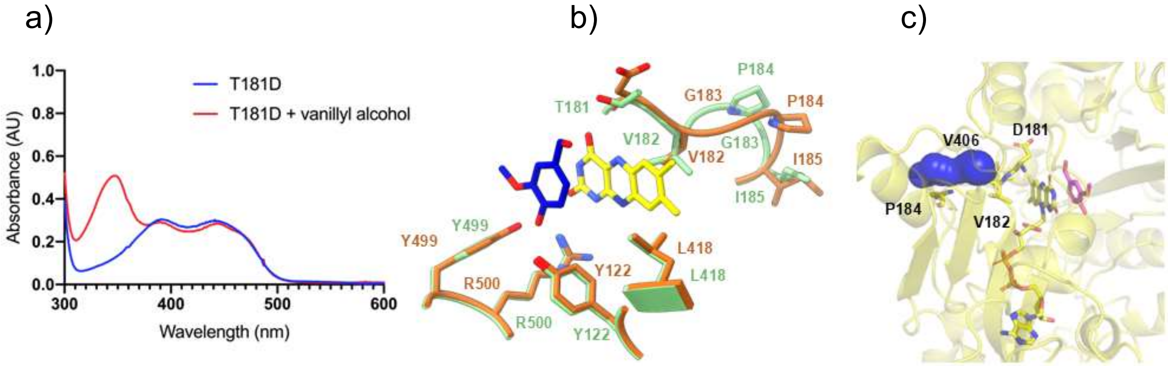

Figure S10. **Properties of nsVAO T181D mutant**. (**a**) Incubation of T181D (30 μM) with vanillyl alcohol (45 μM). The spectra before substrate addition and after 30-minute incubation are in blue and red, respectively. Vanillin, formed upon vanillyl alcohol oxidation, has an absorbance peak at 340 nm (ε=14000 M−1·cm−1). (**b**) Structural comparison between wild type (green carbons) and T181D (dark orange carbons) nsVAOs. (**c**) Accessibility of the flavin N5-C4a locus. Compared to the wild-type nsVAO (**Figure 8c**), there is a narrowing of the tunnel leading to the flavin re side, as revealed by the mutant's crystal structure

Table S1a. Data collection and refinement statistics of VAD: wild type, P151L, P151I and VAD P151V.

	Wild Type	P151L	P151I	P151V
PDB code	8S7P	8S7U	8S7Q	8S7W
Space group	$C222_1$	$P4_12_12$	$P2_12_12_1$	$P2_12_12_1$
Unit cell axes (Å)	90.14, 143.22, 287.75	138.495, 138.495, 180.947	100.443, 113.338, 130.168	100.62, 114.64, 130.71
Unit cell angles (°)	90, 90, 90	90, 90, 90	90, 90, 90	90, 90, 90
Resolution (Å)	67.49– 2.35	47.31– 1.80	52.01– 2.40	49.86– 2.10
R_{merge}	0.166 (3.295)	0.146 (2.885)	0.198 (1.032)	0.159 (1.132)
$CC_{1/2}$	0.998 (0.488)	0.999 (0.390)	0.994 (0.664)	0.989 (0.394)
Completeness (%)	100.0 (99.6)	99.7 (100.0)	99.7 (96.9)	98.8 (99.1)
Unique reflections	77772 (4521)	161652 (7961)	58641 (4368)	87358 (4434)
Multiplicity	21.3 (16.1)	13.5 (13.4)	8.8 (7.8)	4.3 (4.1)
Overall $I/\sigma\ (I)$	12.6 (0.8)	11.8 (0.9)	9.1 (2.1)	8.4 (1.6)
Protein residues	1556	1038	1038	1038
FAD molecules	3	2	2	2
Ligand	-	-	-	-
Water molecules	48	718	358	589
R/R_{free} (%)	22.8 /27.9	15.5/18.0	17.8/23.1	17.2/20.9
Rms bond length	0.001	0.001	0.001	0.001
Rms bond angle	0.518	1.497	0.488	1.343
Ramachandran outliers (%)	0.2	0.1	0.2	0.0
Average B-factor	57.0	30.0	34.0	28.0

Table S1b. Data collection and refinement statistics of VAD: P151L, P151T, P151N and P151G.

	**P151L with eugenol**	**P151T**	**P151N**	**P151G**
PDB code	8S7N	8S7R	8S7T	8S7S
Space group	$P4_12_12$	$C222_1$	$C222_1$	$C222_1$
Unit cell axes (Å)	139.02, 139,02, 181.06	91.949 144.451 286.785	89.525 142.426 286.436	90.804 144.145 289.452
Unit cell angles (°)	90, 90, 90	90, 90, 90	90, 90, 90	90, 90, 90
Resolution (Å)	49.20– 1.80	143.39– 1.90	143.22-2.10	64.60 – 2.50
R_{merge}	0.194 (3.735)	0.066 (1.253)	0.139 (2.349)	0.202 (3.274)
$CC_{1/2}$	0.998 (0.388)	0.991 (0.423)	0.999 (0.553)	0.997 (0.605)
Completeness (%)	100.0 (100.0)	97.8 (98.7)	100.0 (100.0)	100.0 (100.0)
Unique reflections	163464 (8025)	146187 (7204)	106762 (5214)	66056 (4381)
Multiplicity	13.5 (13.3)	3.7 (3.8)	20.1 (20.7)	22.8 (23.6)
Overall $I/\sigma (I)$	11.0 (0.9)	9.2 (1.1)	14.3 (1.5)	12.5 (1.2)
Protein residues	1038	1554	1553	1554
FAD molecules	2	3	3	3
Ligand molecules	2	-	-	-
Water molecules	917	468	292	242
R/R_{free} (%)	15.8 /18.4	19.4/ 23.5	18.4/23.2	19.2/25.2
Rms bond length	0.001	0.001	0.001	0.001
Rms bond angle	1.485	0.456	0.463	0.506
Ramachandran outliers (%)	0.0	0.0	0.1	0.2
Average B-factor	27.0	46.0	50.0	57.0

Table S1c. Data collection and refinement statistics of nsVAO.

	nsVAO with vanillyl alcohol	T181D with vanillin
PDB code	9FFK	9FGE
Space group	$P22_12_1$	$P22_12_1$
Unit cell axes (Å)	90.84, 96.77, 129.11	91.33 97.82 129.61
Unit cell angles (°)	90, 90, 90	90, 90, 90
Resolution (Å)	90.85 – 1.70	78.20 – 1.60
R_{merge}	0.162 (2.466)	0.15 (1.476)
$CC_{1/2}$	0.999 (0.707)	0.998 (0.793)
Completeness (%)	99.7 (99.7)	99.6 (99.3)
Unique reflections	125029 (6098)	152440 (7444)
Multiplicity	20.0 (18.6)	9.3 (9.1)
Overall $I/\sigma (I)$	10.1 (1.3)	8.8 (1.6)
Protein residues	1002	1002
FAD molecules	2	2
Ligand molecules	2	2
Water molecules	558	772
R/R_{free} (%)	22.3 /25.4	17.5/20.4
Rms bond length	0.002	0.001
Rms bond angle	1.449	0.579
Ramachandran outliers (%)	1	0.2
Average B-factor	27.0	18.0

Table S2. Genetic context of VAD from *Marinicaulis flavus* and VAO from *Novosphingobium* sp.

	GenBank Accession Code	Annotation
Upstream genes	WP_104830656.1	UbiD family decarboxylase
	WP_104830657.1	Hypothetical protein
	WP_104830658.1	Pilus assembly protein TadG-related protein
	WP_104830659.1	Pilus assembly protein
	WP_104830660.1	Pilus assembly protein
	WP_207764815.1	Putative cytochrome c
VAD	WP_104830661.1	FAD-binding oxidoreductase
Downstream genes	WP_133162311.1	Hypothetical protein
	WP_104830663.1	ATPase
	WP_104830664.1	Hypothetical protein
	WP_104830665.1	Hypothetical protein
	WP_104830666.1	Glutathione S-transferase family protein
	WP_104830667.1	Glutathione-dependent disulfide-bond oxidoreductase

	GenBank Accession Code	Annotation
Upstream genes	WP_124809343.1	Glycogen/starch/alpha-glucan phosphorylase
	WP_124809344.1	1,4-alpha-glucan branching protein GlgB
	WP_124809345.1	Glucose-1-phosphate adenylyltransferase
	WP_124809346.1	Glycogen synthase GlgA
	WP_124809347.1	Alpha-D-glucose phosphate-specific phosphoglucomutase
	WP_223806624.1	Glycogen debranching protein GlgX
nsVAO	WP_124809348.1	FAD-binding oxidoreductase
Downstream genes	WP_124809349.1	Putative cytochrome c
	WP_190287233.1	TetR/AcrR family transcriptional regulator
	WP_124809351.1	Alpha/beta hydrolase
	WP_124809352.1	DUF2334 domain-containing protein
	WP_223806625.1	Hypothetical protein
	WP_124809353.1	Glycosyltransferase

Table S3. **Steady state kinetic parameters of P151L VAD**

		O_2		
**VAD**	**Substrate**	**k_{cat} (s^{-1})**	**K_M (µM)**	**k_{cat}/K_M (s^{-1} mM^{-1})**
P151L	Vanillyl alcohol	0.83±0.05	131.9±30.4	6
P151L	Eugenol	0.92±0.04	55±8.8	17
P151L	4-Hydroxy benzylalcohol	1.02±0.03	86.74±9.1	12
P151L	4-Methoxy methylphenol	0.38±0.02	600±70	0.6

Measured at 25 °C in 50 mM potassium phosphate pH 7.5, 150 mM NaCl. Data are shown as average values ± standard deviation. n = 3 independent experiments. No activity was detected on *p*-cresol.

Supplementary Table 4. Primers used for quick change mutagenesis

Primer name	Sequence
VAD_P151Lfw	gtgccggacctgggctggggtagtgtggtcgg
VAD_P15Lrv	accccagcccaggtccggcacatcgatccata
VAD_P151Vfw	ccggacgtgggctggggtagtgtg
VAD_P151Vrv	ccagcccacgtccggcacatcgatc
VAD_P151Ifw	ccggacattggctggggtagtgtg
VAD_P151Irw	ccagccaatgtccggcacatcgatc
VAD_P151Gfd	gtgccggacggcggctggggtagtgtggtcgg
VAD_P151Grv	accccagccgccgtccggcacatcgatccata
VAD_P151Nfw	gtgccggacaacggctggggtagtgtggtcgg
VAD_P151Nrv	accccagccttggtccggcacatcgatccata
VAD_P151Tfw	ccggacaccggctggggtagtgtg
VAD_P151Trv	ccagccggtgtccggcacatcgatc
nsVAO_T181Dfw	acgtcccagatgtcggcccgattgcatctccag
nsVAO_T181Drv	cgggccgacatctgggacgtcaatccaaaggga

EUGO_L152Pfw	gacccgggatggggcagcgtg
EUGO_L152Prv	ccatcccgggtcgggacagtc
